# Supplementary material for: Synthesis of Fluorogenic Arylureas and Amides and Their Interaction with Amines: A Competition between Turn-on Fluorescence and Organic Radicals on the Way to a Smart Label for Fish Freshness
Source: Molecules. 2021 Mar 5;26(5):1404. doi: 10.3390/molecules26051404 (PMC7961427; doi:10.3390/molecules26051404)
Supplement: Supplementary file 1 [file molecules-26-01404-s001.pdf]

Supporting Information

Synthesis of Fluorogenic Arylureas and Amides, and Their Interaction with Amines: A Competition Between Turn-on Fluorescence and Organic Radicals on the Way to a Smart Label for Fish Freshness.

Javier García-Tojal, José V. Cuevas, M. Josefa Rojo, Borja Díaz de Greñu, Carla Hernando-Muñoz, José García-Calvo, Mateo M. Salgado, Tomás Torroba*

Department of Chemistry, Faculty of Science, University of Burgos, 09001 Burgos, Spain. ttorroba@ubu.es

Characterization of all compounds……………………………………………………………S2-S21

Additional EPR measurements…………………………………………………………………S22-S25

UV-Vis Titrations…………………………………………………………………………………S26-S27

Fluorescence measurements……………………………………………………………………S28-S30

Detection of biogenic amines…………………………………………………………………S31-S32

Quantum chemical calculations………………………………………………………………S33-S37

Characterization of all compounds:

Synthesis of 5-(4-aminophenyl)indan-1-one:

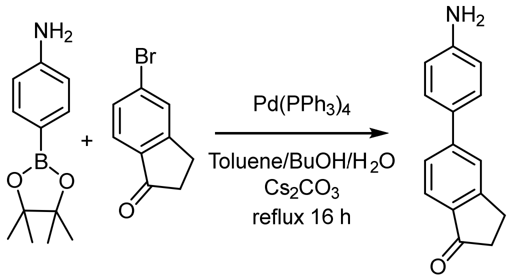

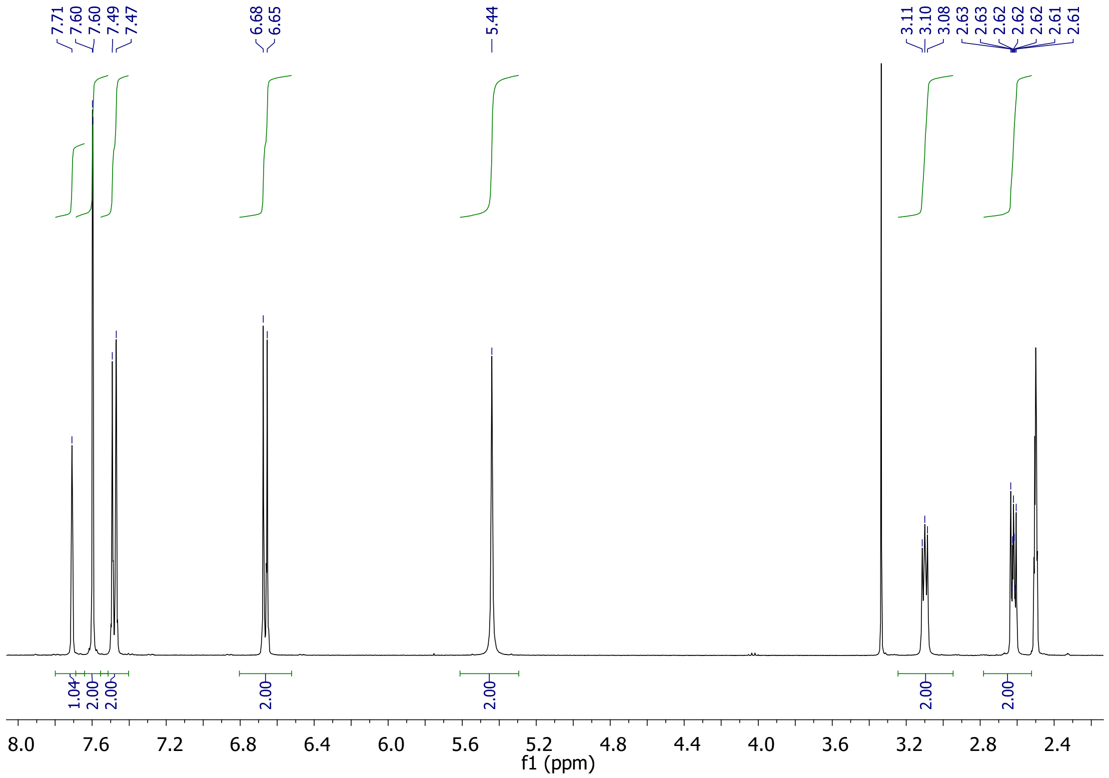

Figure S1: ^{1}H NMR (DMSO-d_6, 400 MHz) of 5-(4-aminophenyl)indan-1-one

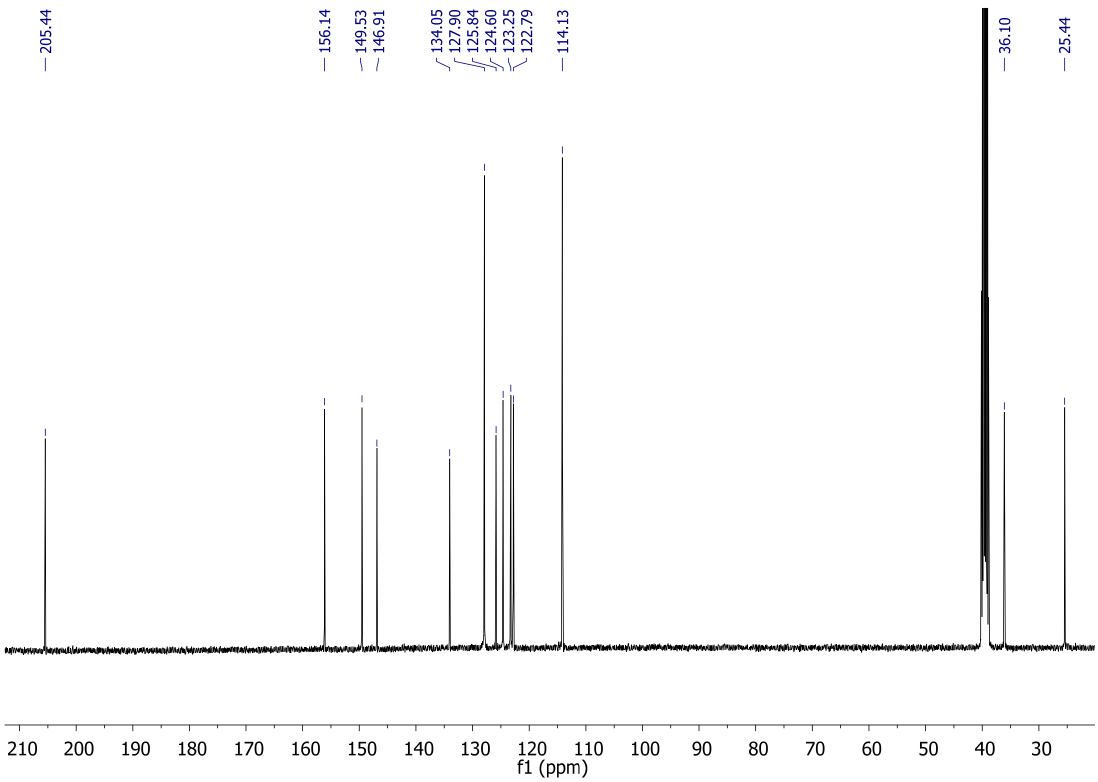

Figure S2: ^{13}C NMR (DMSO-*d*$_6$, 100 MHz) of 5-(4-aminophenyl)indan-1-one

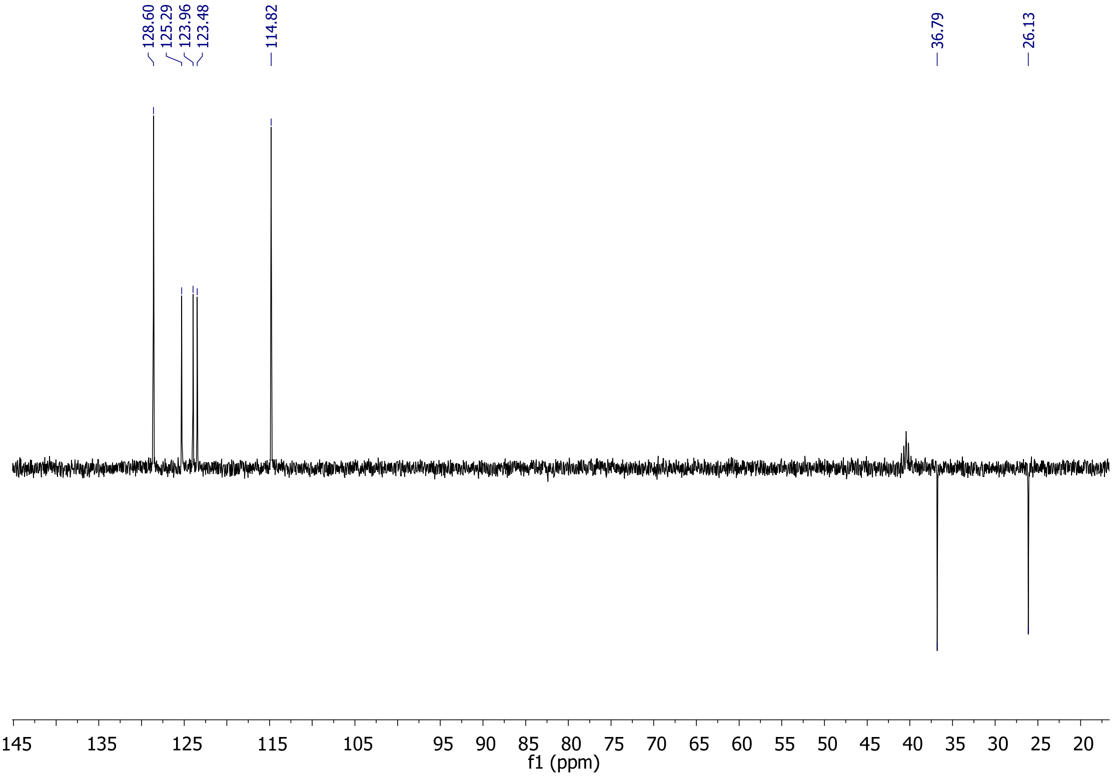

Figure S3: DEPT (DMSO-*d*$_6$, 100 MHz) of 5-(4-aminophenyl)indan-1-one

Synthesis of 1-dicyanomethylene-5-(4-aminophenyl)indane:

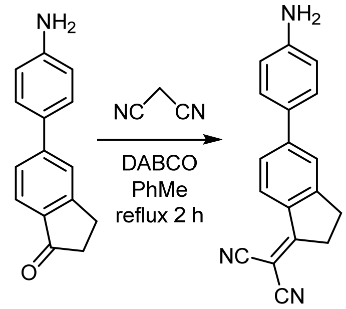

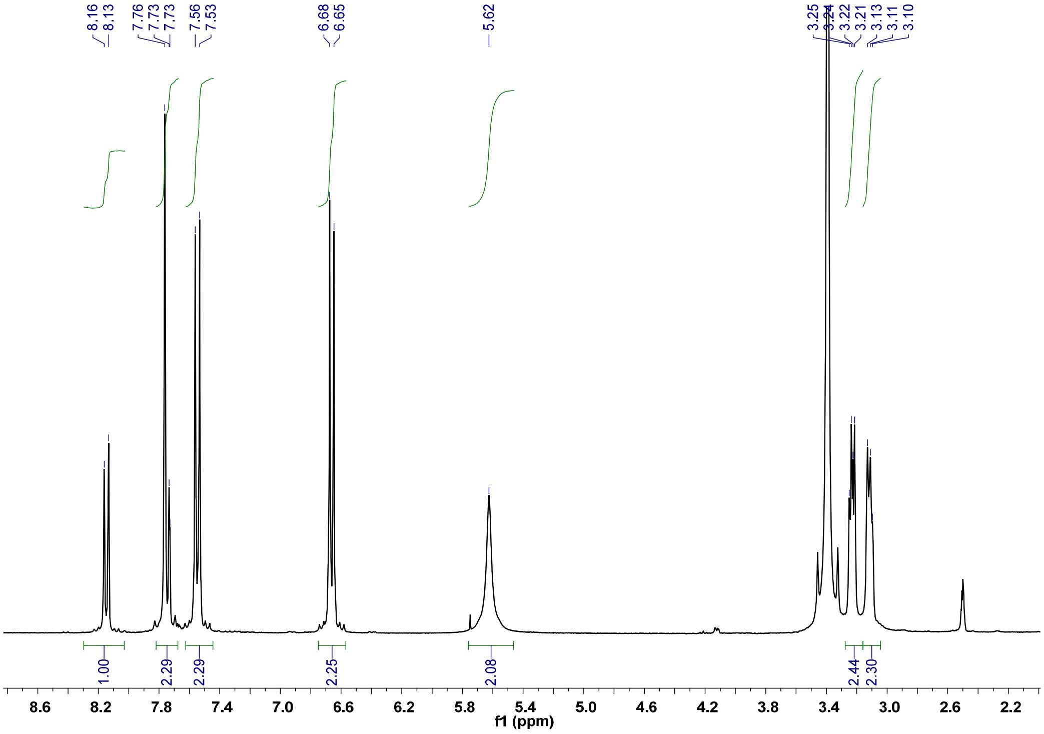

Figure S4: ^{1}H NMR (DMSO-d_6, 300 MHz) of 1-dicyanomethylene-5-(4-aminophenyl)indane

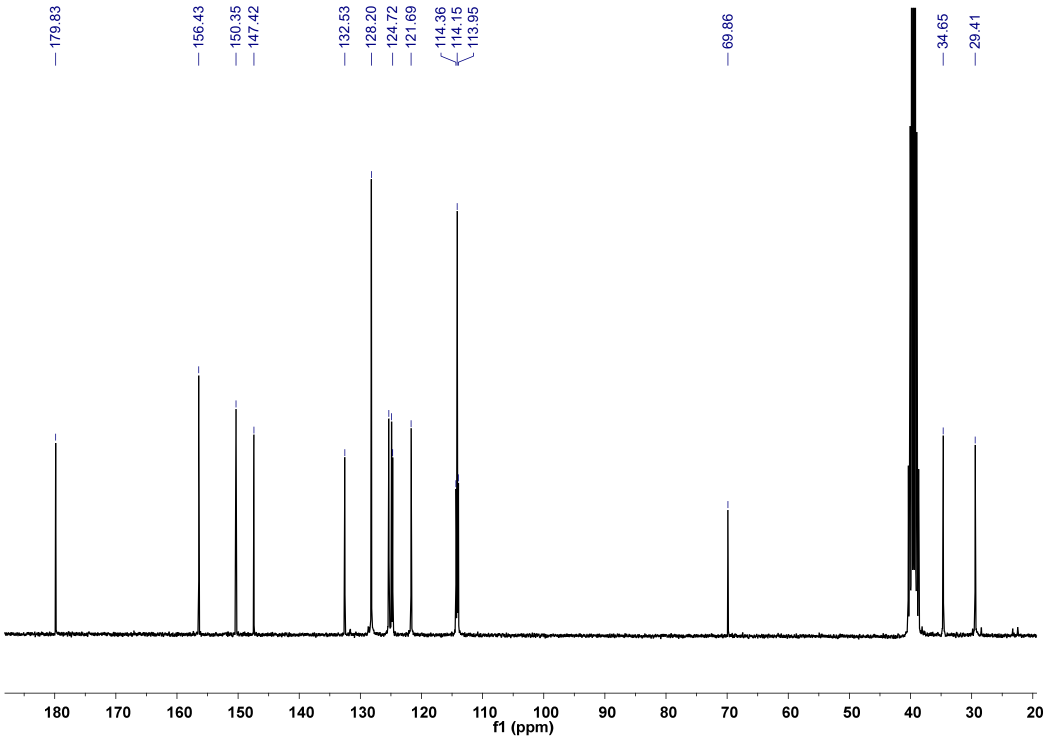

Figure S5: ^{13}C NMR (DMSO-d_6, 75 MHz) of 1-dicyanomethylene-5-(4-aminophenyl)indane

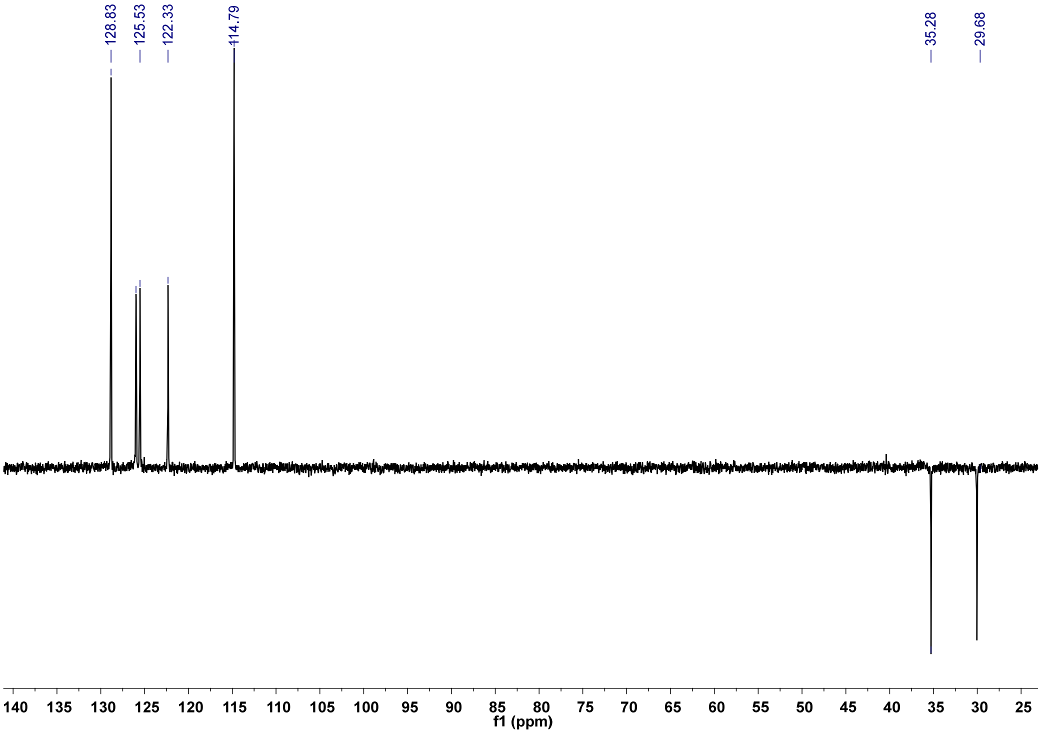

Figure S6: DEPT (DMSO-d_6, 75 MHz) of 1-dicyanomethylene-5-(4-aminophenyl)indane

Synthesis of 1-(4-methoxyphenyl-3-[4-(1-(dicyanomethyleneindan-5-yl)phenyl]urea 1:

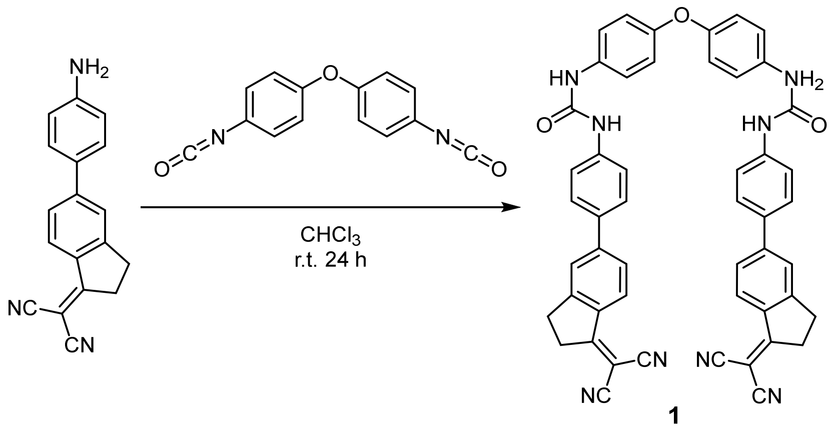

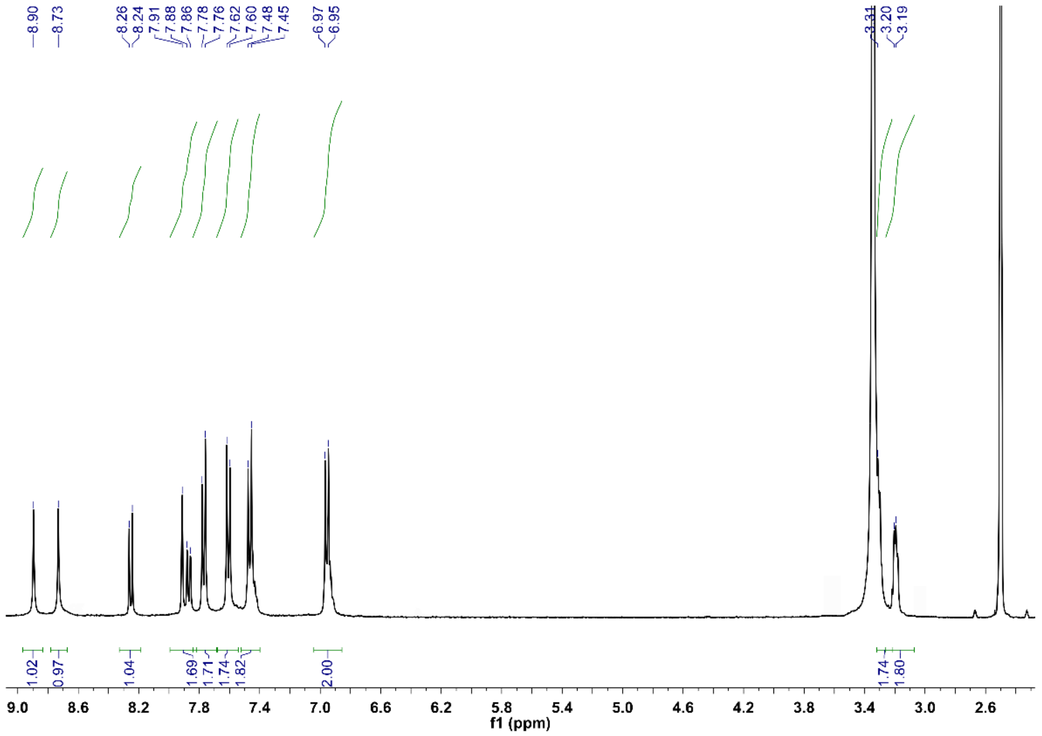

Figure S7: ^{1}H NMR (DMSO-d_6, 400 MHz) of 1-(4-methoxyphenyl-3-[4-(1-(dicyanomethyleneindan-5-yl)phenyl]urea 1

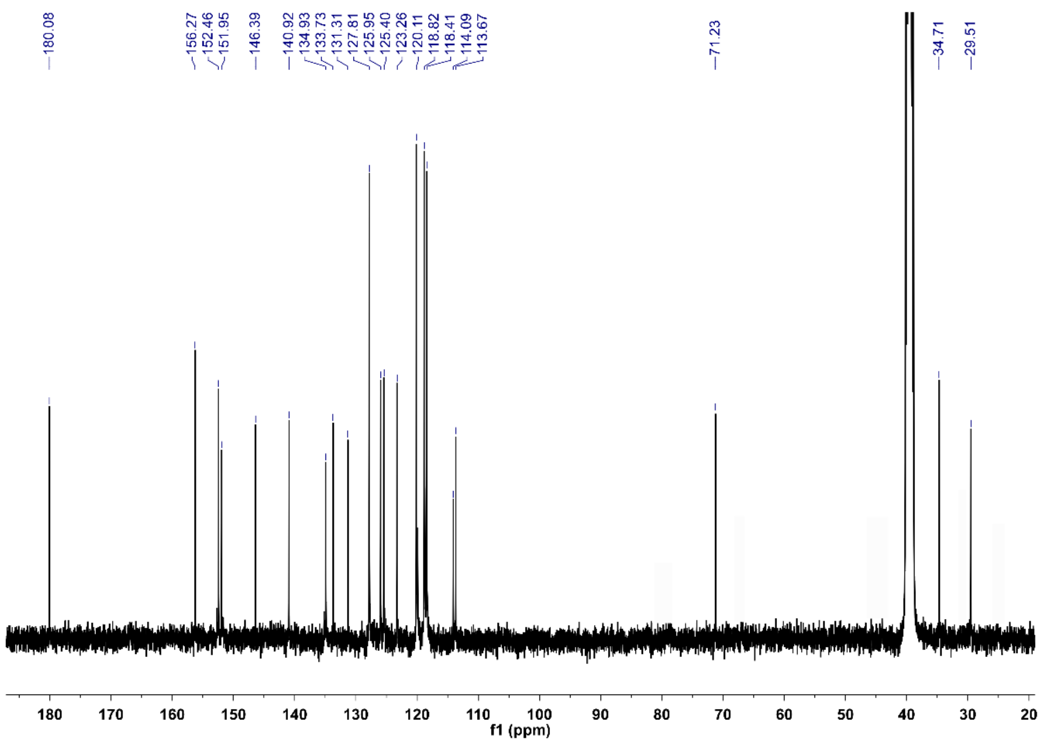

Figure S8: ^{13}C NMR (DMSO-d_6, 100 MHz) of 1-(4-methoxyphenyl-3-[4-(1-(dicyanomethyleneindan-5-yl)phenyl]urea 1

Synthesis of 1,3-bis[4-(1-dicyanomethyleneindan-5-yl)phenylureidomethyl)-benzene 2:

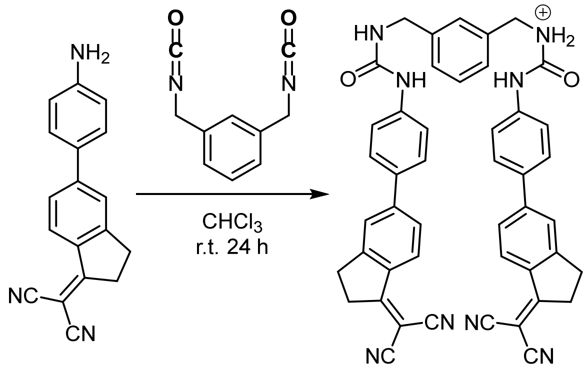

2

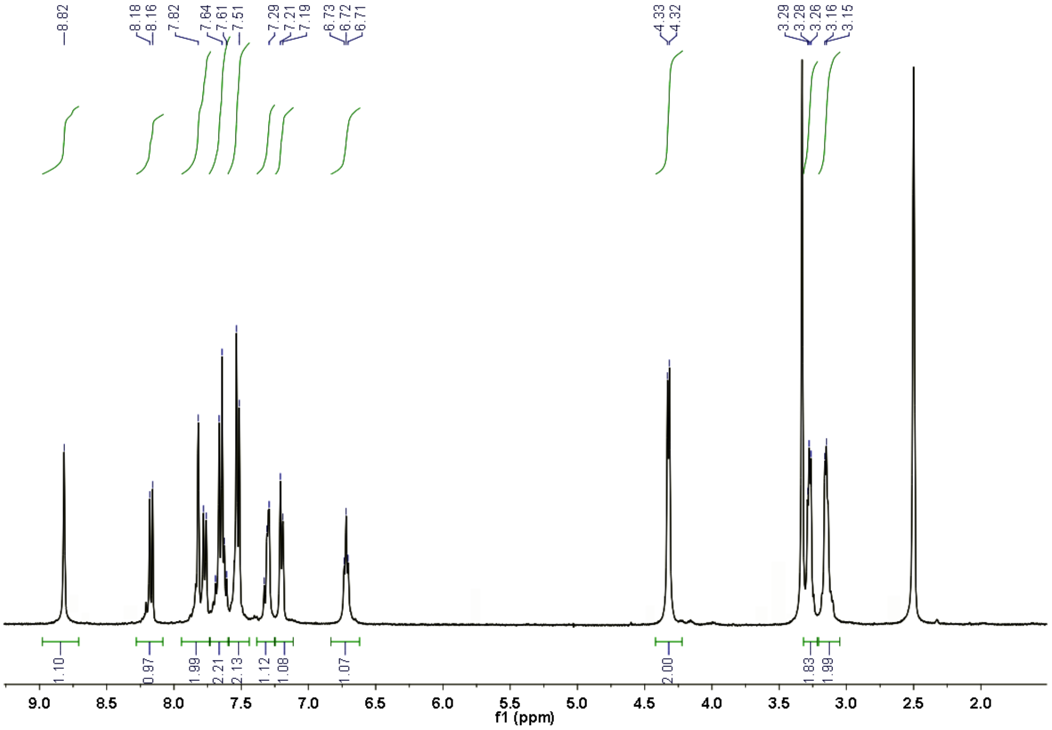

Figure S9: ^{1}H NMR (DMSO-*d₆*, 400 MHz) of 1,3-bis[4-(1-dicyanomethyleneindan-5-yl)phenylureidomethyl)-benzene 2

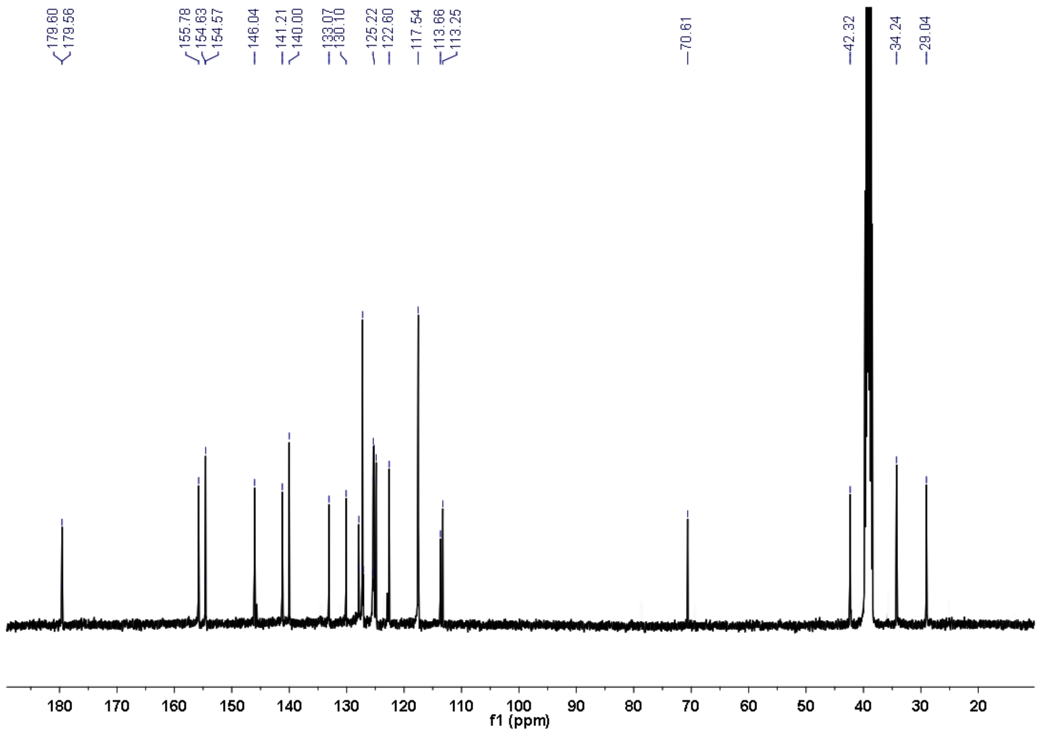

Figure S10: ^{13}C NMR (DMSO-d_6, 100 MHz) of 1,3-bis[4-(1-dicyanomethyleneindan-5-yl)phenylureidomethyl)-benzene 2

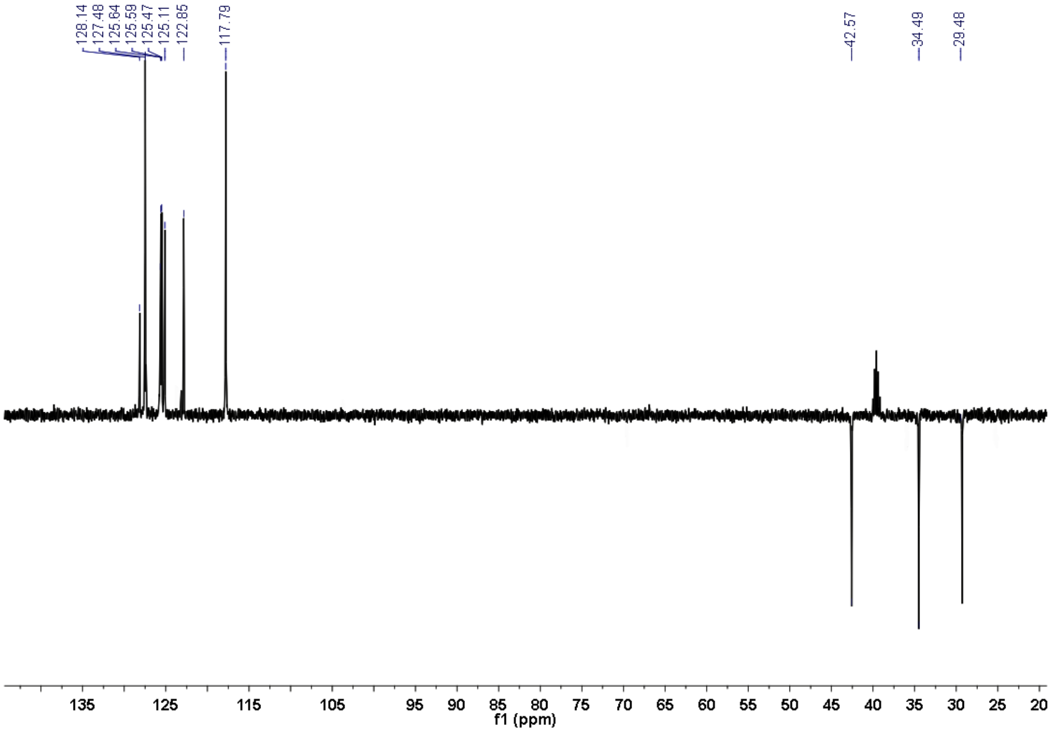

Figure S11: DEPT (DMSO-d_6, 100 MHz) of 1,3-bis[4-(1-dicyanomethyleneindan-5-yl)phenylureidomethyl)-benzene 2

Synthesis of 1-(4-methoxyphenyl-3-[4-(1-(dicyanomethyleneindan-5-yl)phenyl]urea 3:

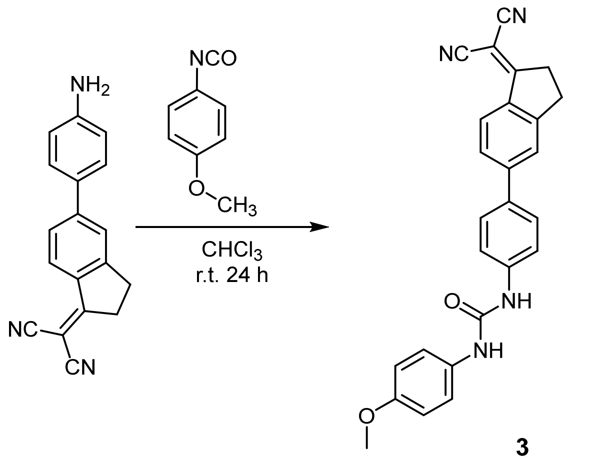

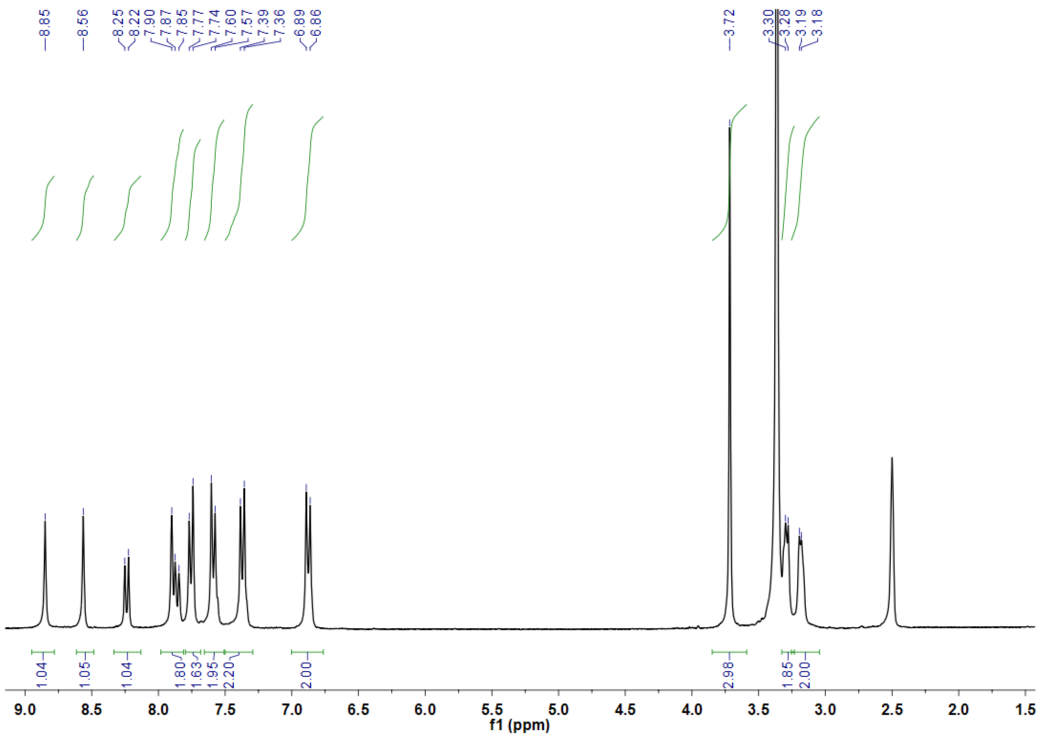

Figure S12: ^{1}H NMR (DMSO-d_6, 300 MHz) of 1-(4-methoxyphenyl-3-[4-(1-(dicyanomethyleneindan-5-yl)phenyl]urea 3

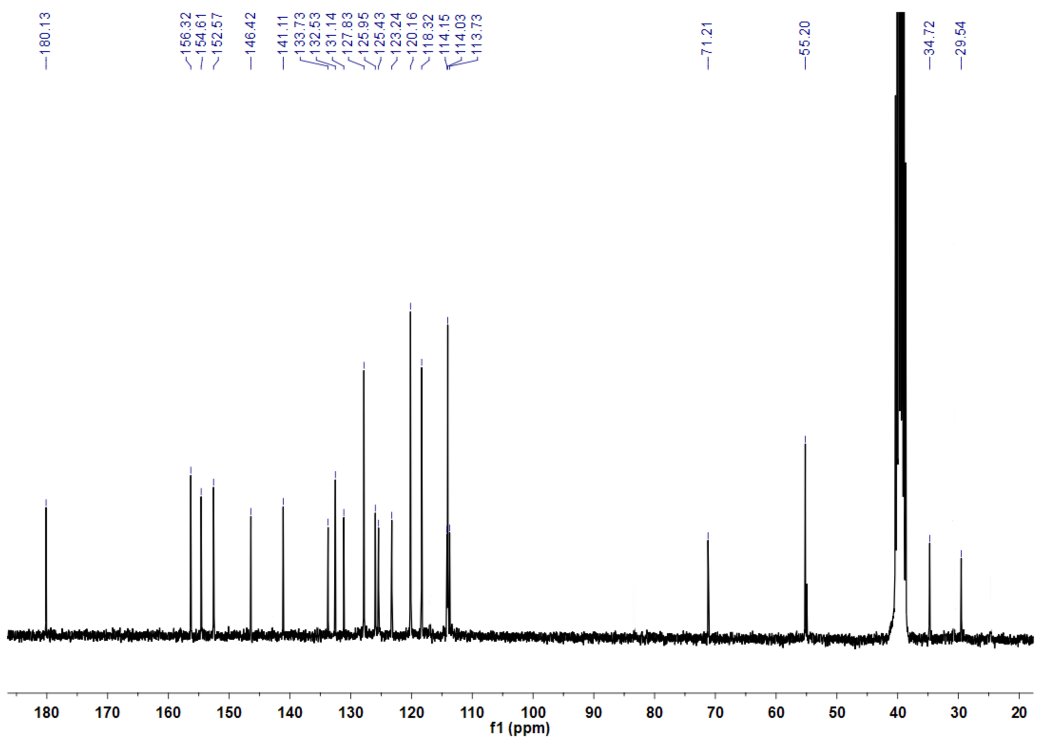

Figure S13: ^{13}C NMR (DMSO-d_6, 75 MHz) of 1-(4-methoxyphenyl-3-[4-(1-(dicyanomethyleneindan-5-yl)phenyl]urea 3

Synthesis of 1-[4-(*N,N*-dimethylamino)phenyl]-3-[4-(1-(dicyanomethylene-indan-5-yl)phenyl]urea 4:

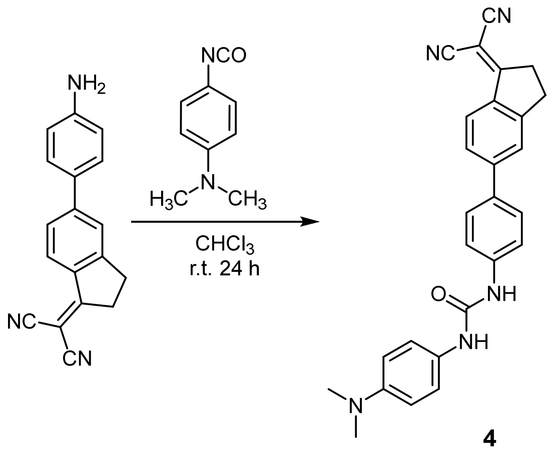

4

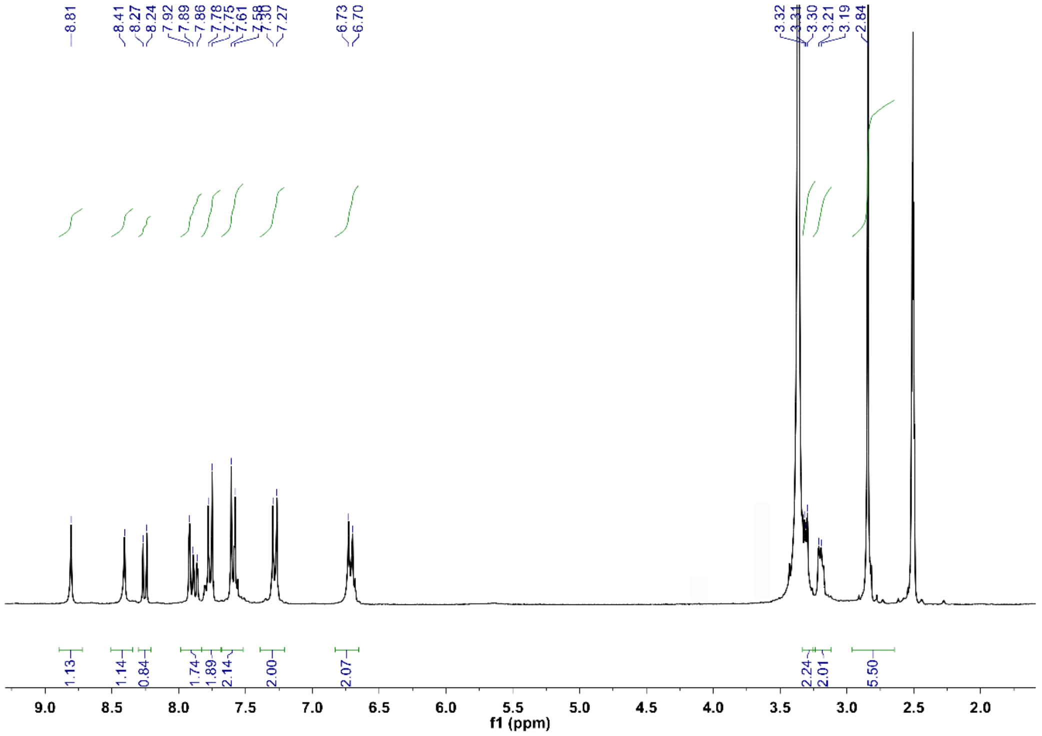

Figure S14: ^{1}H NMR (DMSO-*d*6, 300 MHz) of 1-[4-(*N,N*-dimethylamino)phenyl]-3-[4-(1-(dicyanomethylene-indan-5-yl)phenyl]urea 4

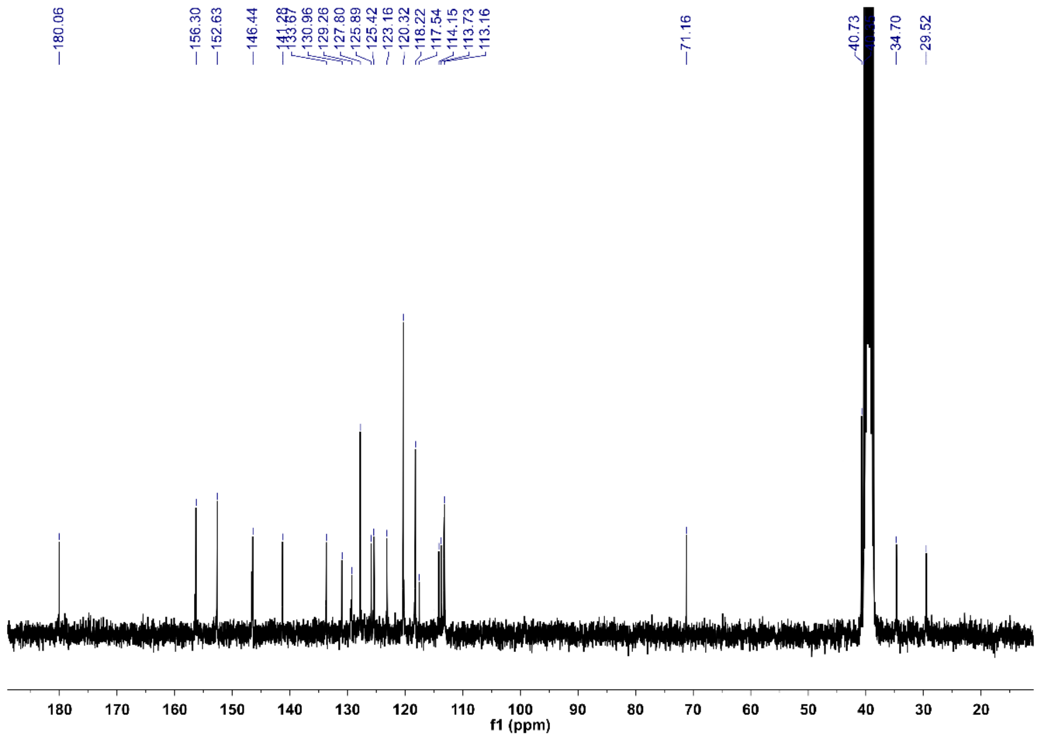

Figure S15: ^{13}C NMR (DMSO-*d*$_6$, 75 MHz) of 1-[4-(*N,N*-dimethylamino)phenyl]-3-[4-(1-(dicyanomethylene-indan-5-yl)phenyl]urea 4

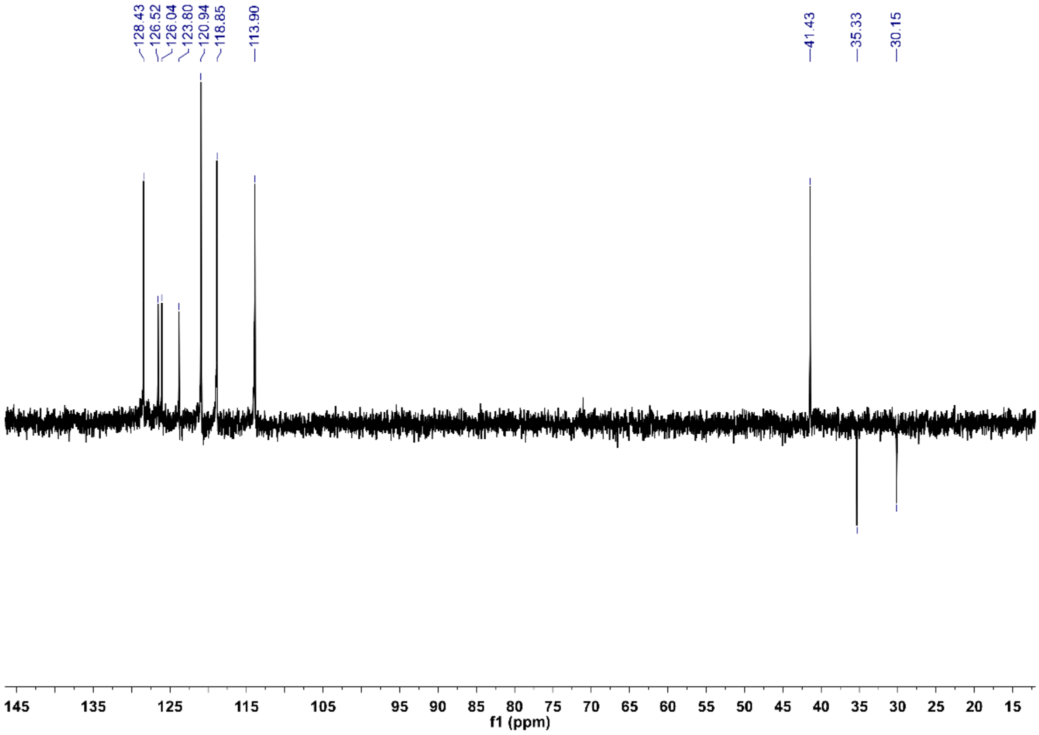

Figure S16: DEPT (DMSO-*d*$_6$, 75 MHz) of 1-[4-(*N,N*-dimethylamino)phenyl]-3-[4-(1-(dicyanomethylene-indan-5-yl)phenyl]urea 4

Synthesis of 1-(4-methylthiophenyl)-3-[4-(1-(dicyanomethyleneindan-5-yl)-phenyl]urea 5:

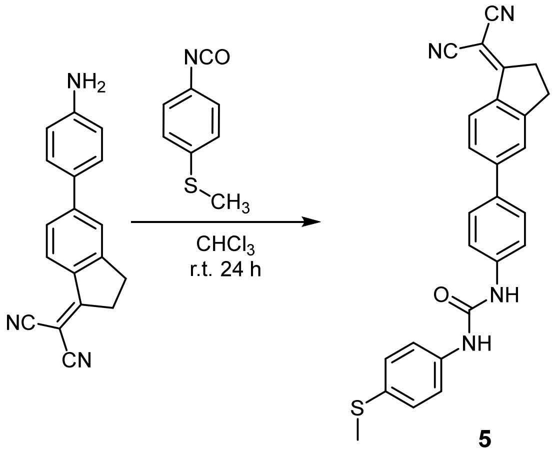

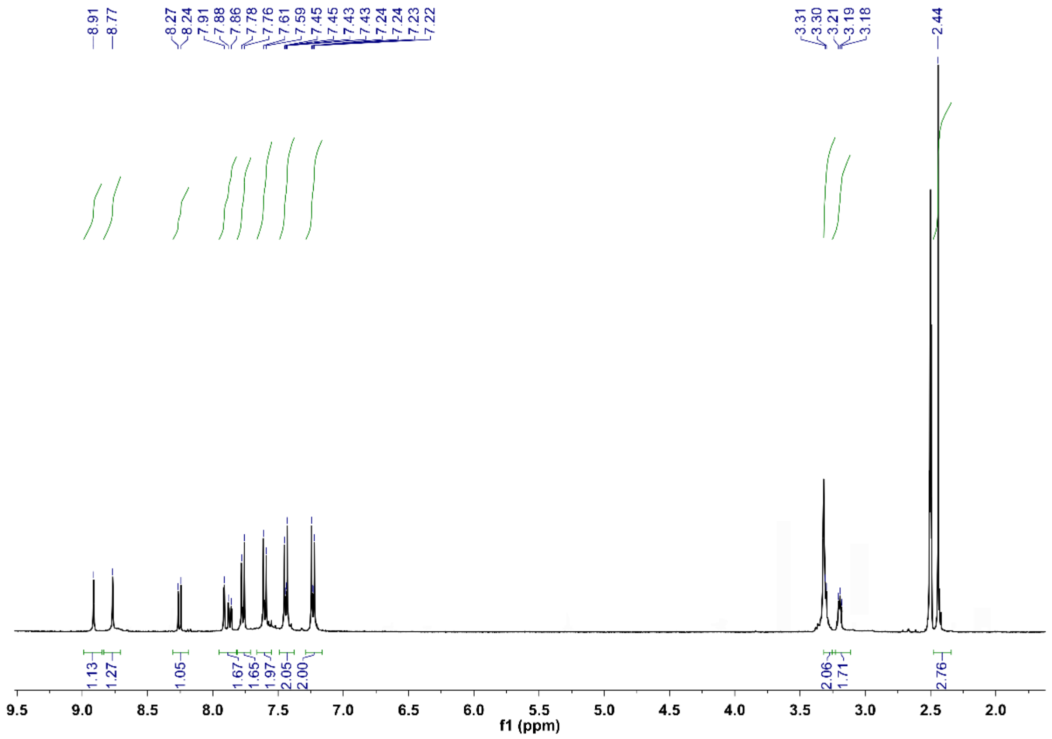

Figure S17: ^{1}H NMR (DMSO-d_6, 300 MHz) of 1-(4-methylthiophenyl)-3-[4-(1-(dicyanomethyleneindan-5-yl)-phenyl]urea 5

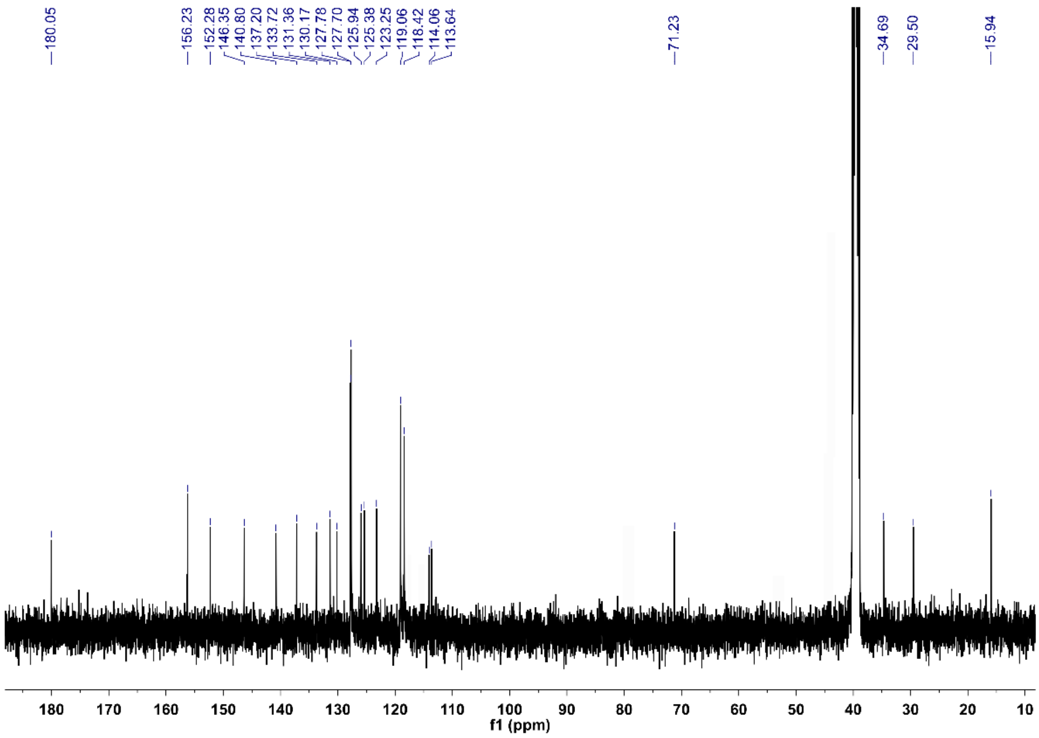

Figure S18: ^{13}C NMR (DMSO-d_6, 75 MHz) of 1-(4-methylthiophenyl)-3-[4-(1-(dicyanomethyleneindan-5-yl)-phenyl]urea 5

Synthesis of *N,N*-bis-[4-(1-(dicyanomethyleneindan-5-yl)phenyl]pyridine-2,6-dicarboxamide 6:

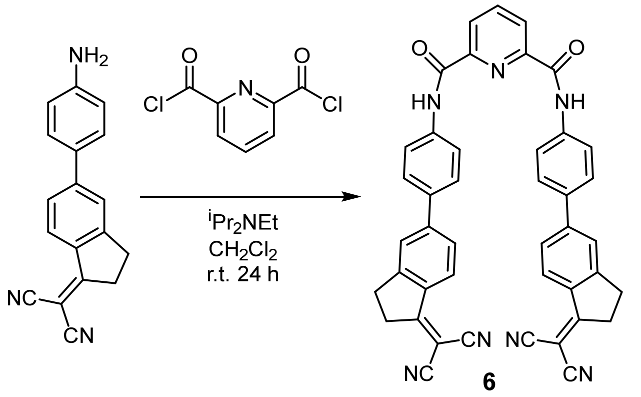

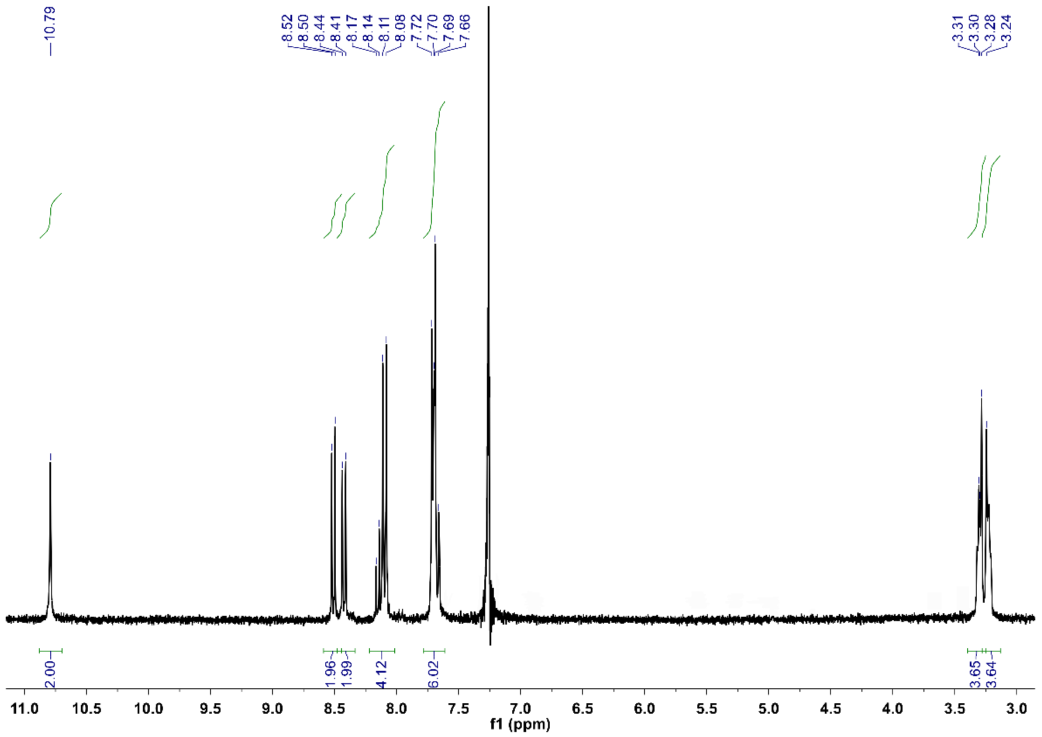

Figure S19: ^{1}H NMR (CDCl$_3$, 400 MHz) of *N,N*-bis-[4-(1-(dicyanomethyleneindan-5-yl)phenyl]pyridine-2,6-dicarboxamide 6

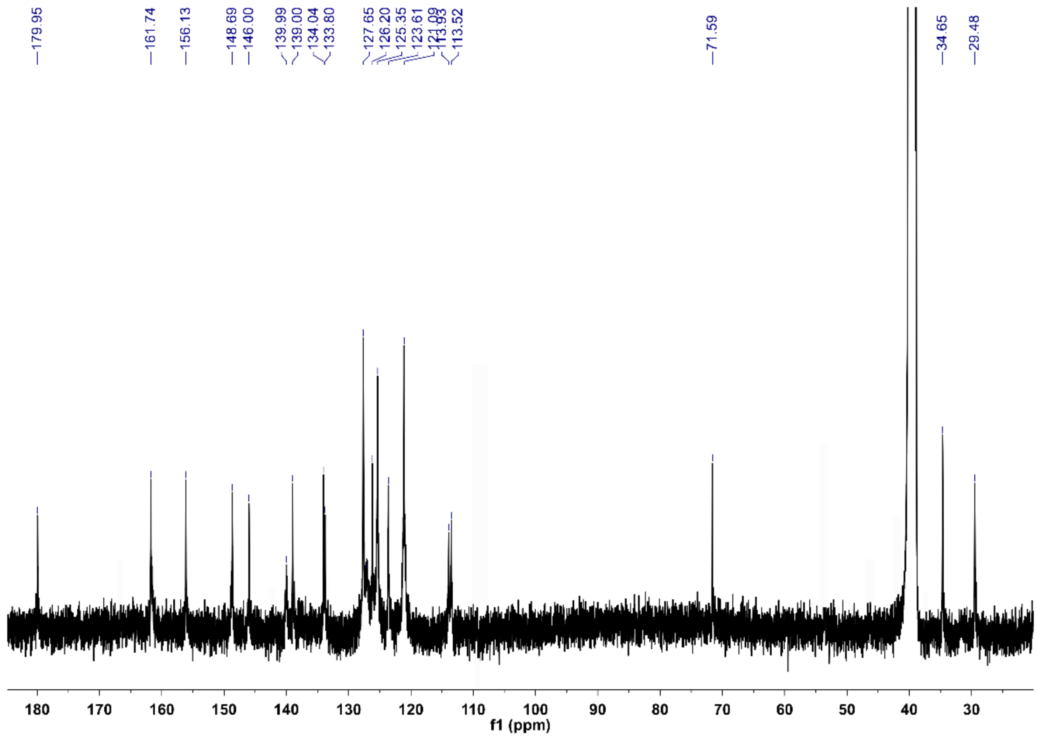

Figure S20: ^{13}C NMR (DMSO-d_6, 75 MHz) of *N,N*-bis-[4-(1-(dicyanomethyleneindan-5-yl)phenyl]pyridine-2,6-dicarboxamide 6

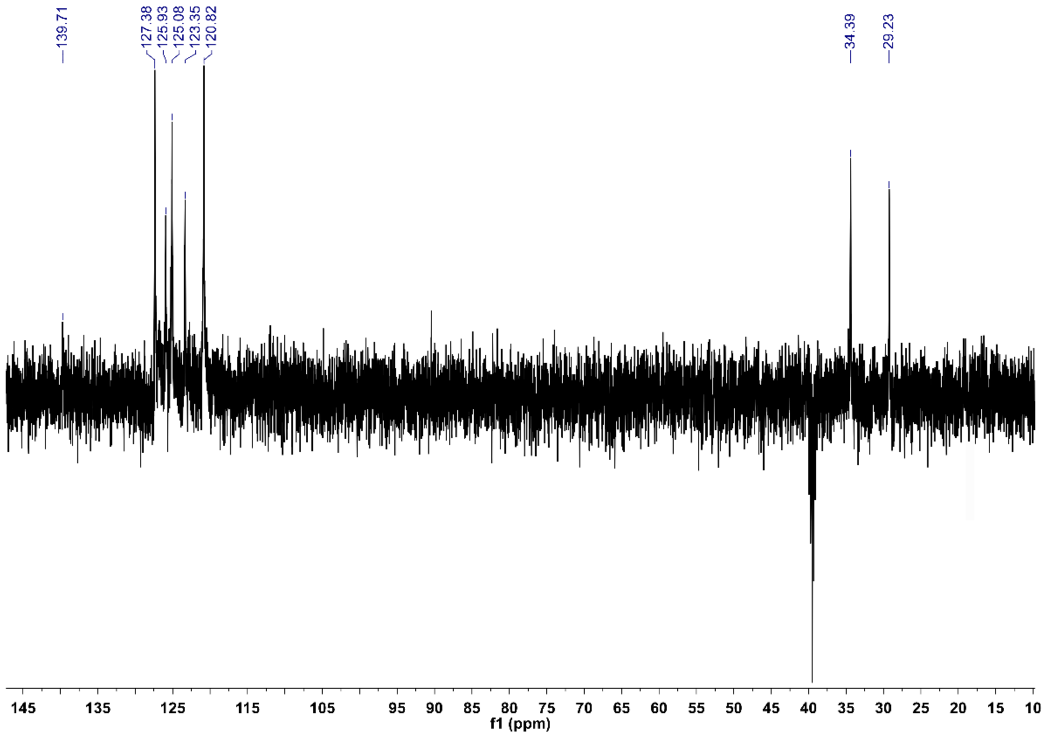

Figure S21: DEPT (DMSO-d_6, 75 MHz) of *N,N*-bis-[4-(1-(dicyanomethyleneindan-5-yl)phenyl]pyridine-2,6-dicarboxamide 6

Synthesis of 6-(piperidin-1-yl)-[3,4'-bipyridine]-2',6'-dicarboxylic acid

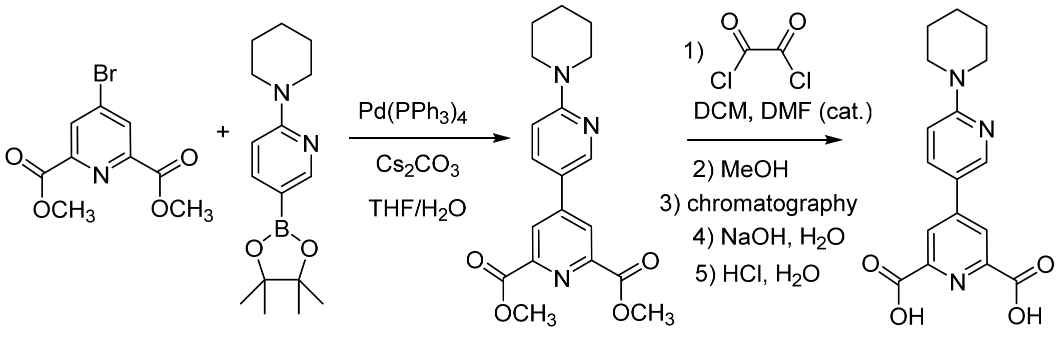

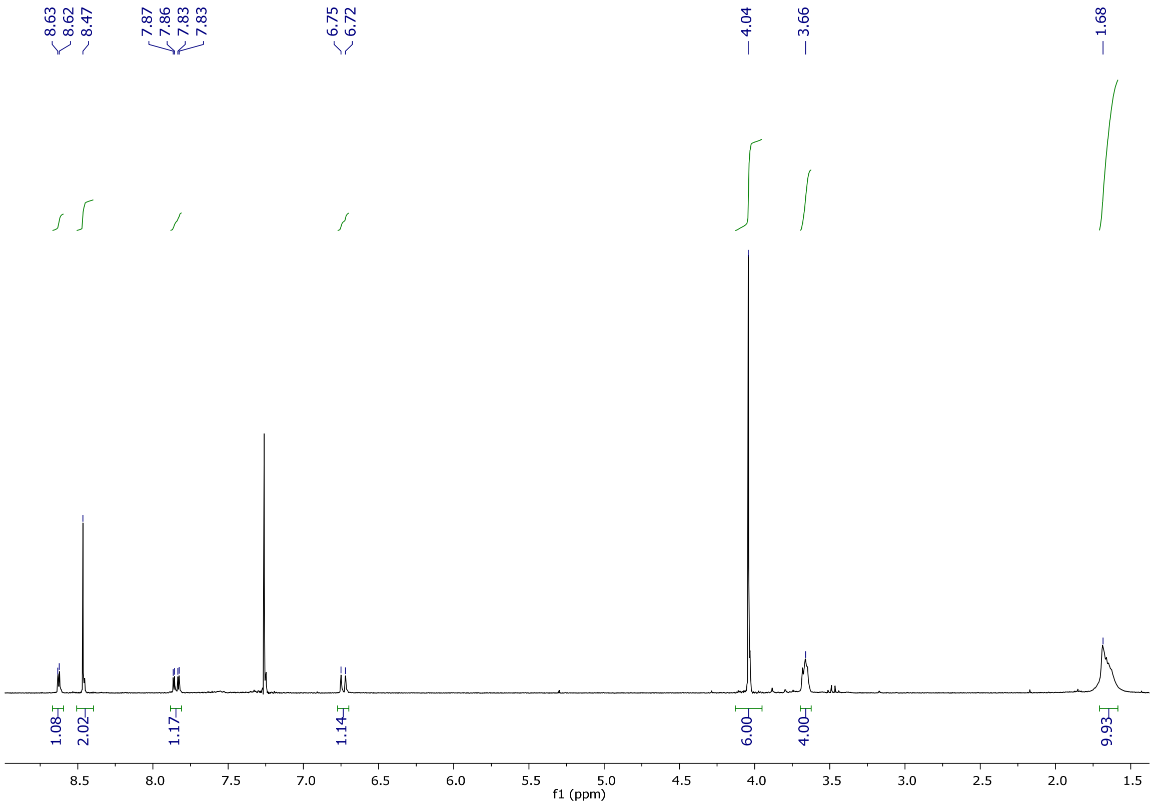

Figure S22: ^{1}H-NMR ($CDCl_3$, 300 MHz) of dimethyl 6-(piperidin-1-yl)-[3,4'-bipyridine]-2',6'-dicarboxylate

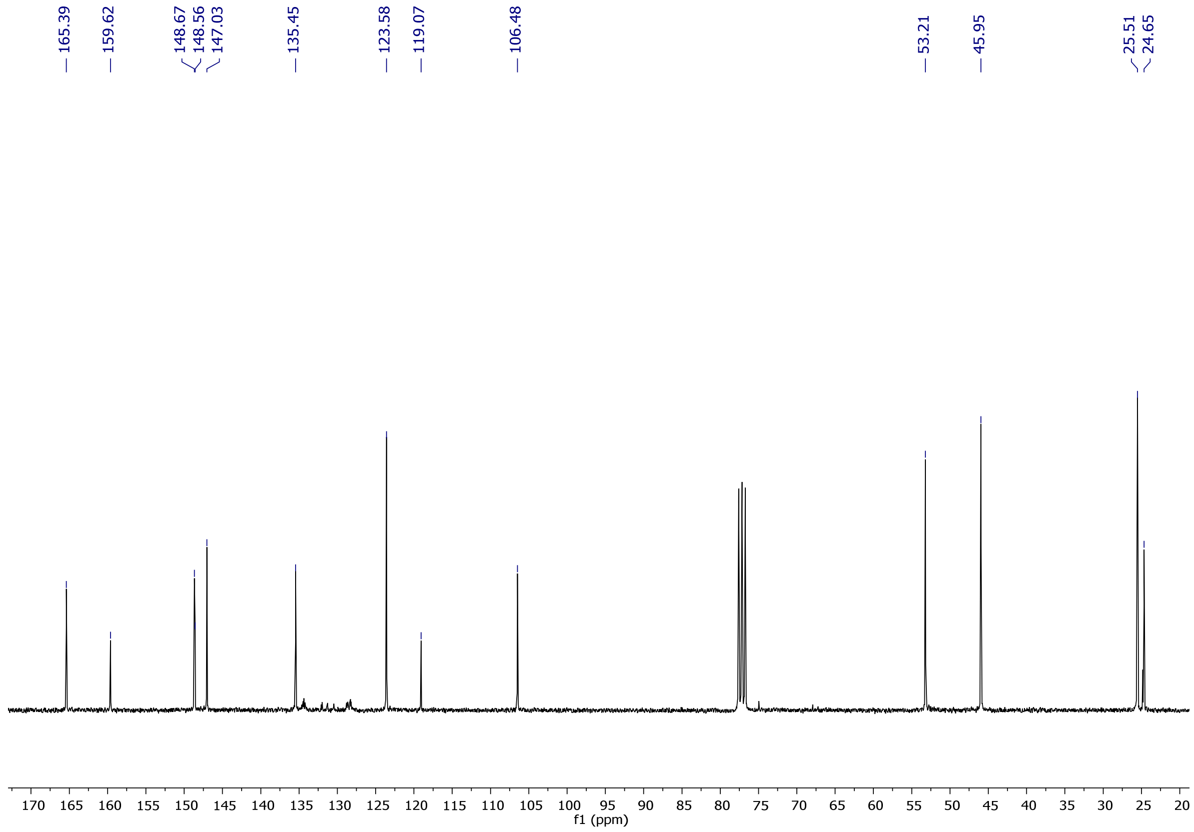

Figure S23: ^{13}C-NMR ($CDCl_3$, 75 MHz) of dimethyl 6-(piperidin-1-yl)-[3,4'-bipyridine]-2',6'-dicarboxylate

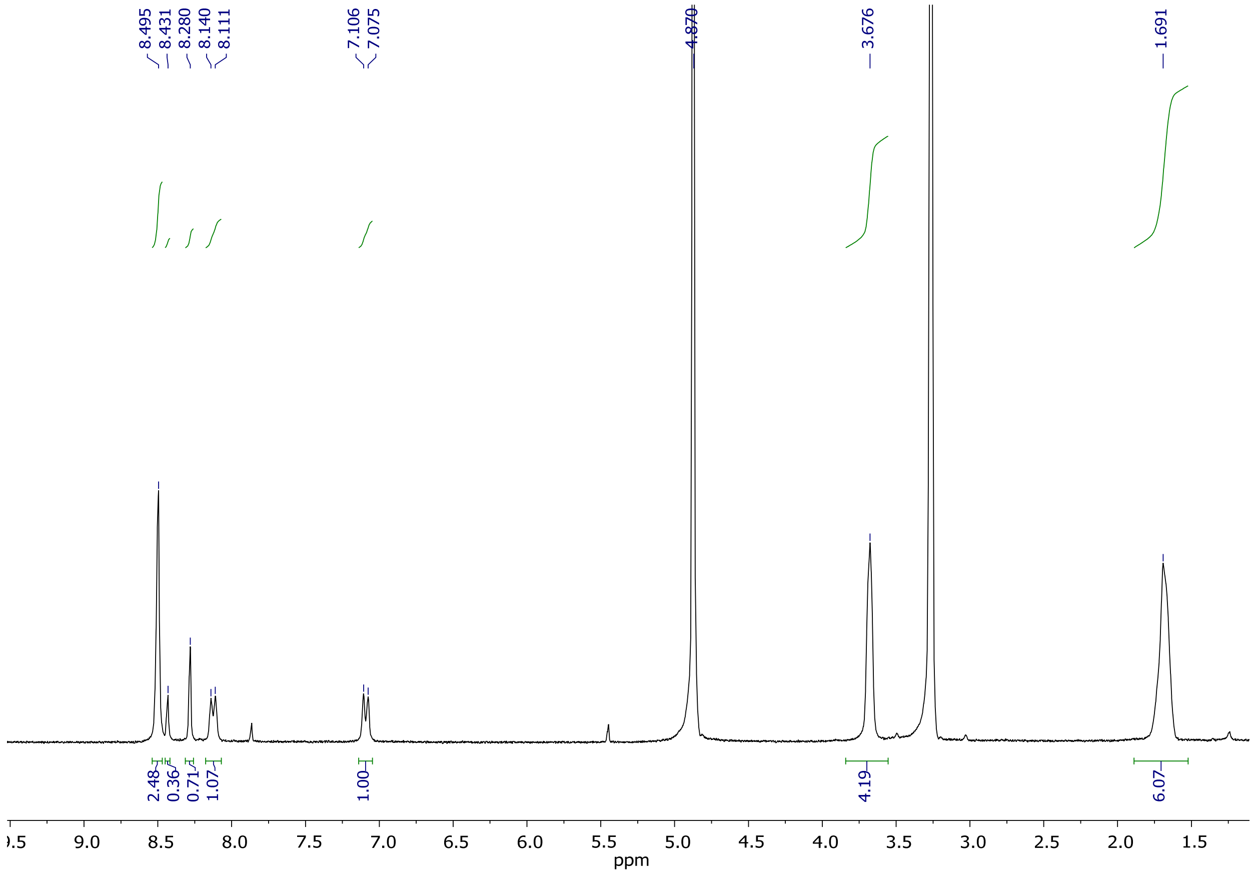

Figure S24: ^{1}H-NMR (CD_3OD, 300 MHz) of 6-(piperidin-1-yl)-[3,4'-bipyridine]-2',6'-dicarboxylic acid.

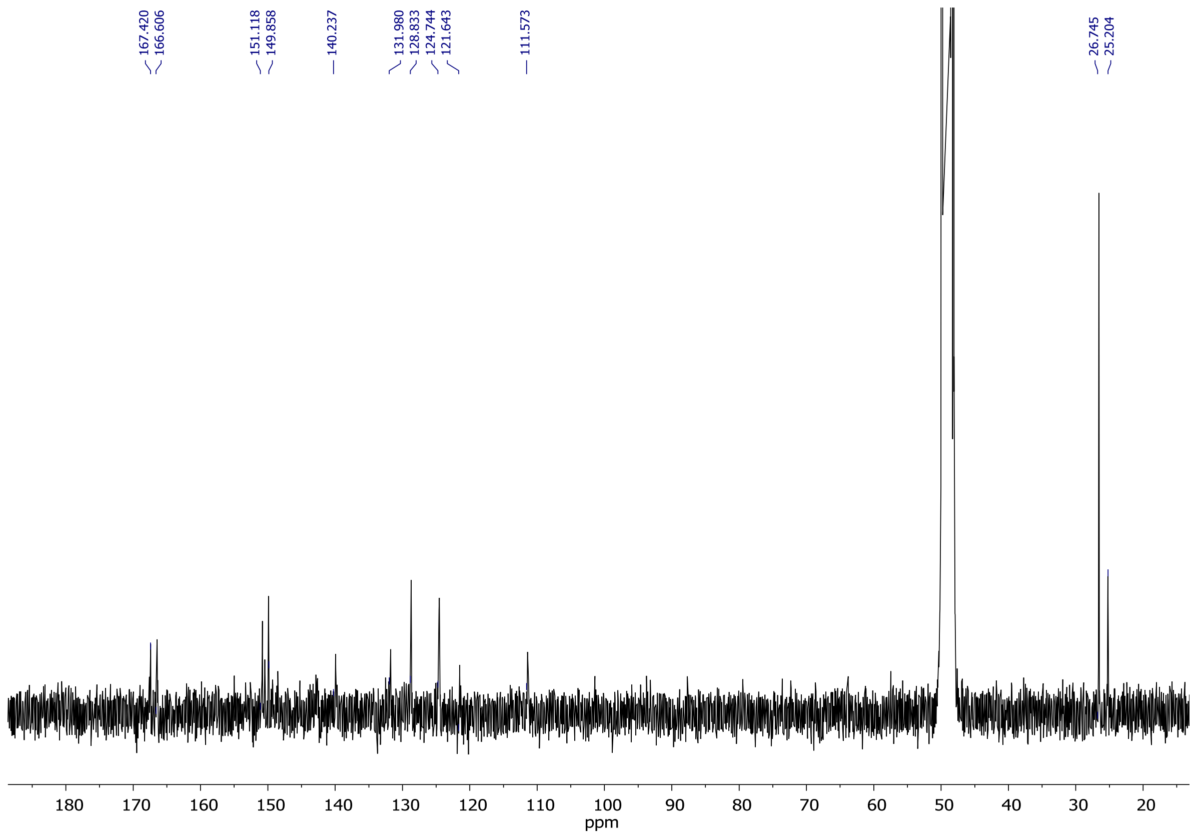

Figure S25: ^{13}C-NMR (CD$_3$OD, 75 MHz) of 6-(piperidin-1-yl)-[3,4'-bipyridine]-2',6'-dicarboxylic acid.

Synthesis of 5,5'-(((6-cyclohexyl-[3,4'-bipyridine]-2',6'-dicarbonyl)bis(piperazine-4,1-diyl))bis(pyridine-6,3-diyl))bis-(indan-1-one) 7:

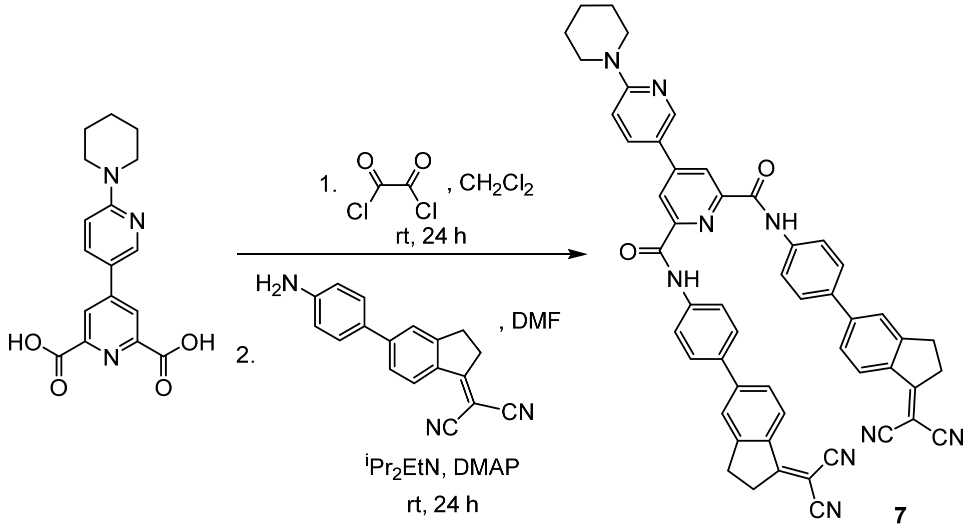

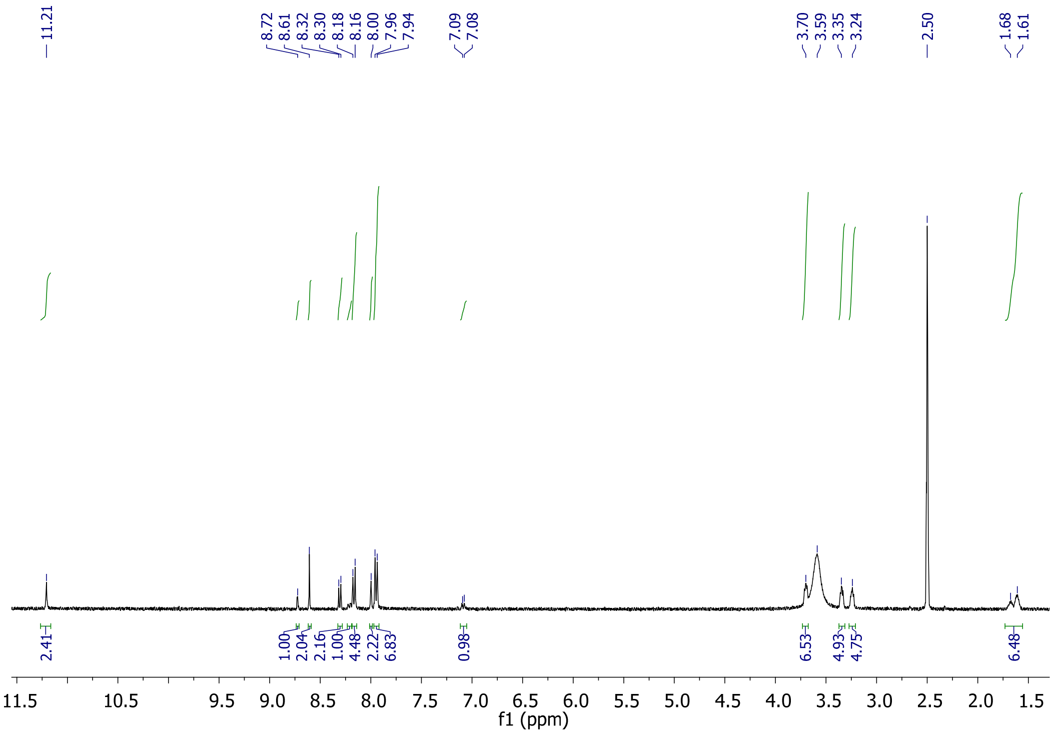

Figure S26: ^{1}H-NMR (DMSO-*d*$_6$, 400 MHz) of 5,5'-(((6-cyclohexyl-[3,4'-bipyridine]-2',6'-dicarbonyl)bis(piperazine-4,1-diyl))bis(pyridine-6,3-diyl))bis(indan-1-one) 7

Additional EPR measurements:

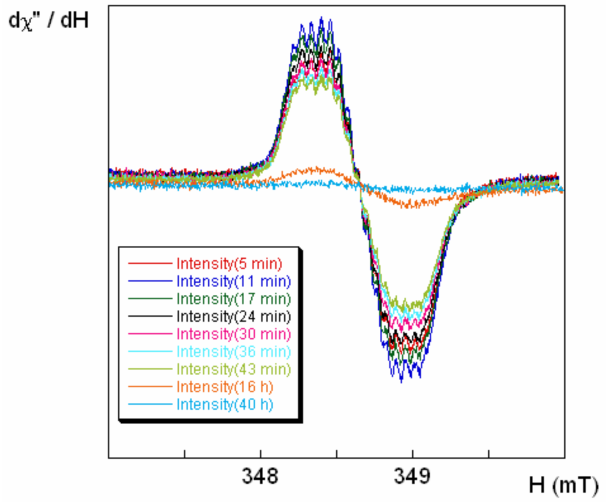

Figure S27. Evolution with time of the EPR spectra in the interaction of 1 and pyrrolidine at room temperature. The experiment starts when 100 μl of a 10^{-2} M solution of pyrrolidine in DMSO were added over a mixture prepared 1 h before that contains 100 μl of a 10^{-2} M solution of **1** in DMSO, 100 μl of water, 100 μl of acetone and 100 μl of DMSO. After shaking, 200 μl of the mixture were pipetted into a flat cell. Experimental details of the data collection: modulation amplitude 0.1 mT, time constant 40.96 ms, conversion time 327.68 ms, gain 6.32 10^4, power 20 mW and microwave frequency 9.7410 (5 min), 9.7410 (11 min), 9.7410 (17 min), 9.7410 (24 min), 9.7460 (30 min), 9.7410 (36 min), 9.7410 (43 min), 9.7410 (16 h), 9.7760 (40 h) GHz.

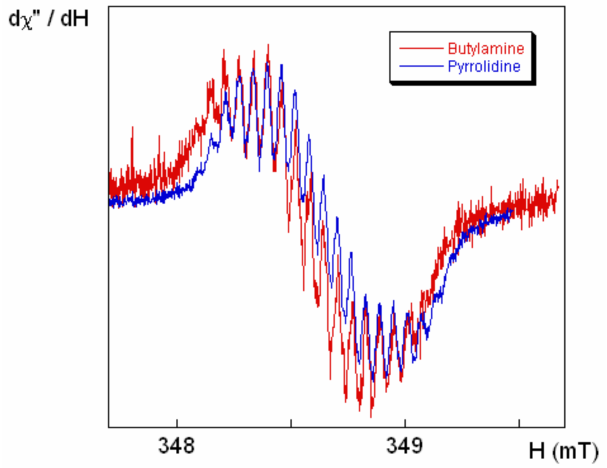

Figure S28. Comparison between the EPR spectra of 6 after addition of pyrrolidine or butylamine (10^{-2} M solution in DMSO). The experimental conditions are the same given for the pyrrolidine in Figure S27. The microwave frequency in the case of the butylamine is 9.7750 GHz.

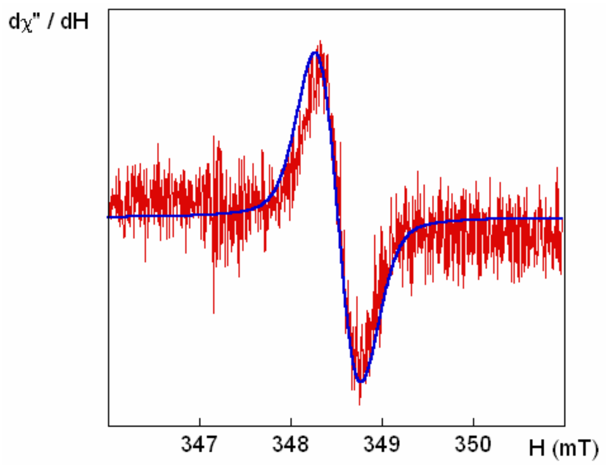

Figure S29. Room-temperature X-band EPR spectrum of 1 after addition of DABCO (in red) and the best fit (in blue). Experimental details: 100 µl of a 10^{-2} M solution of **1** in DMSO, 100 µl of water, 100 µl of acetone and 100 µl of DMSO were allowed to mix for 220 min. Then, 100 µl of a 10^{-2} M solution of DABCO in DMSO were added. After shaking, 200 µl of the mixture were pipetted into a flat cell. Data collection: modulation amplitude 0.1 mT, time constant 40.96 ms, conversion time 327.68 ms, gain 6.32 10^4, power 20 mW and microwave frequency 9.7729 GHz.

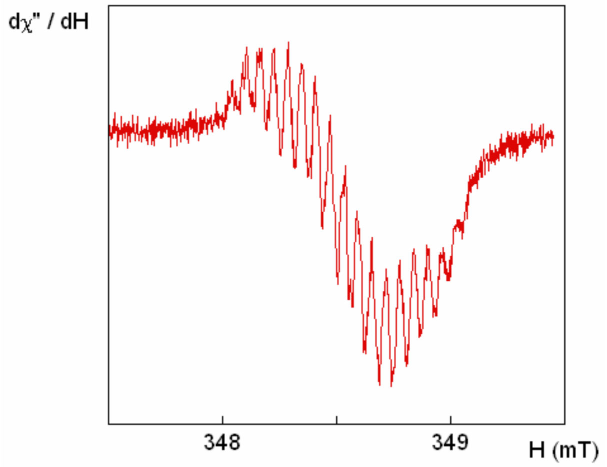

Figure S30. EPR spectrum of the interaction of 1 with tetrabutylammonium cyanide. Experimental details: 100 µl of a 10^{-2} M solution of **1** in DMSO, 100 µl of water, 100 µl of acetone and 100 µl of DMSO were allowed to mix for 220 min. Then, 100 µl of a 10^{-2} M solution of tetrabutylammonium cyanide in DMSO were added. After shaking, 200 µl of the mixture were pipetted into a flat cell. The present spectrum was recorded 28 min after the beginning of the experiment. Data collection: modulation amplitude 0.02 mT, time constant 40.96 ms, conversion time 327.68 ms, gain 6.32 10^4, power 20 mW and microwave frequency 9.7737 GHz.

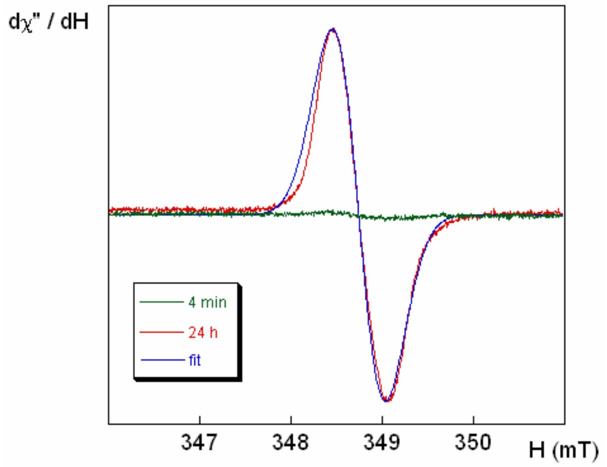

Figure S31. Evolution with time of the EPR spectra in the interaction of 1 and an aqueous solution of NaOH 1 M at room temperature. The fit for the 24 h experiment is given in blue. The experiment starts when 50 µl of aqueous NaOH 1 M were added over a mixture prepared 2 h before that contains 100 µl of a 10^{-2} M solution of **1** in DMSO, 50 µl of water, 100 µl

of acetone and 200 µl of DMSO. After shaking, 200 µl of the mixture were pipetted into a flat cell. Experimental details of the data collection: modulation amplitude 0.1 mT, time constant 40.96 ms, conversion time 327.68 ms, gain 6.32 10^4, power 20 mW and microwave frequency 9.7771 (4 min), 9.7763 (24 h) GHz.

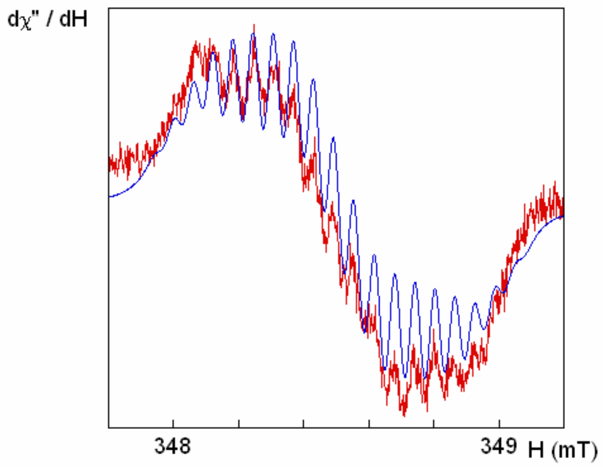

Figure S32. EPR spectrum of the interaction of 2 with pyrrolidine. In blue is given the fit described in text (see Figure S27). Experimental details: 100 µl of a 10^{-2} M solution of **2** in DMSO, 100 µl of water and 100 µl of DMSO were allowed to mix for 1 h. Then, 100 µl of a 10^{-2} M solution of pyrrolidine in DMSO were added. After shaking, 200 µl of the mixture were pipetted into a flat cell. The present spectrum was recorded 14 min after the beginning of the experiment. Data collection: modulation amplitude 0.02 mT, time constant 40.96 ms, conversion time 327.68 ms, gain 6.32 10^4, power 20 mW, the spectrum is averaged from 3 scans and microwave frequency 9.7726 GHz.

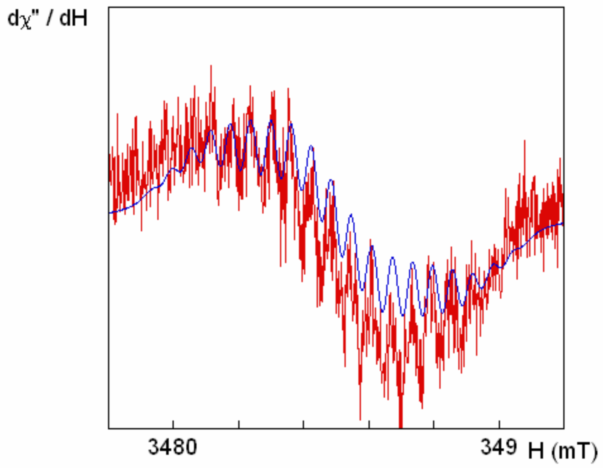

Figure S33. EPR spectrum of the interaction of 3 with pyrrolidine. In blue is given the fit described in text (see Figure S27). Experimental details: 100 µl of a 10^{-2} M solution of **3** in DMSO, 100 µl of water and 100 µl of DMSO were allowed to mix for 1 h. Then, 100 µl of a 10^{-2} M solution of pyrrolidine in DMSO were added. After shaking, 200 µl of the mixture were pipetted into a flat cell. The present spectrum was recorded 10 min after the beginning of the experiment. Data collection: modulation amplitude 0.02 mT, time constant 40.96 ms, conversion time 327.68 ms, gain 6.32 10^4, power 20 mW, the spectrum is averaged from 3 scans and microwave frequency 9.7724 GHz.

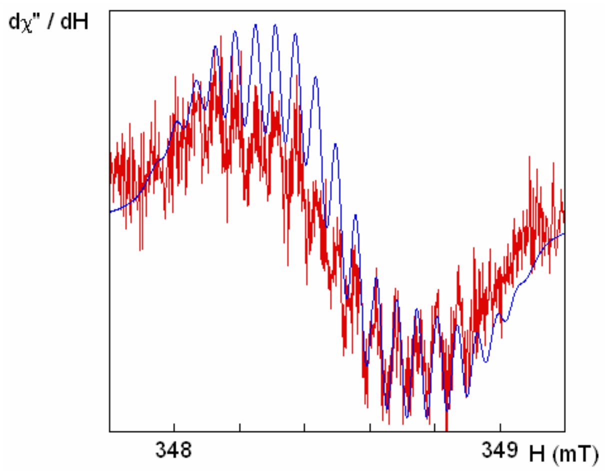

Figure S34. EPR spectrum of the interaction of 4 with pyrrolidine. In blue is given the fit described in text (see Figure S27). Experimental details: 100 µl of a 10^{-2} M solution of **4** in DMSO, 100 µl of water and 100 µl of DMSO were allowed to mix for 1 h. Then, 100 µl of a 10^{-2} M solution of pyrrolidine in DMSO were added. After shaking, 200 µl of the mixture were pipetted into a flat cell. The present spectrum was recorded 10 min after the beginning of the experiment. Data collection: modulation amplitude 0.02 mT, time constant 40.96 ms, conversion time 327.68 ms, gain 6.32 10^4, power 20 mW, the spectrum is averaged from 3 scans and microwave frequency 9.7727 GHz.

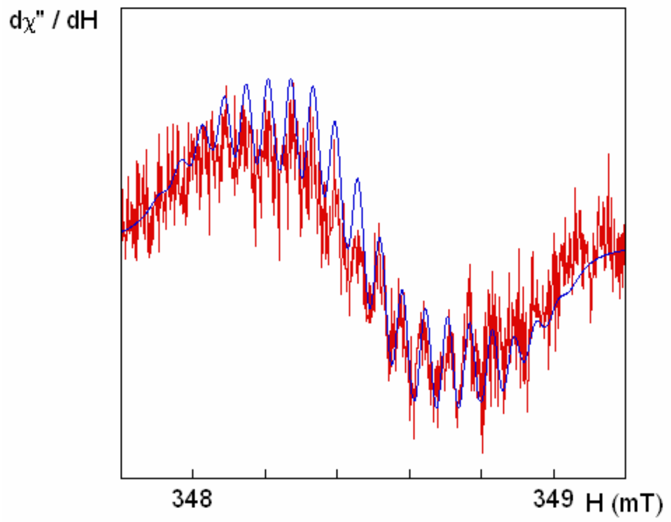

Figure S35. EPR spectrum of the interaction of 5 with pyrrolidine. In blue is given the fit described in text (see Figure S27). Experimental details: 100 µl of a 10^{-2} M solution of **5** in DMSO, 100 µl of water and 100 µl of DMSO were allowed to mix for 1 h. Then, 100 µl of a 10^{-2} M solution of pyrrolidine in DMSO were added. After shaking, 200 µl of the mixture were pipetted into a flat cell. The present spectrum was recorded 11 min after the beginning of the experiment. Data collection: modulation amplitude 0.02 mT, time constant 40.96 ms, conversion time 327.68 ms, gain 6.32 10^4, power 20 mW, the spectrum is averaged from 3 scans and microwave frequency 9.7716 GHz.

UV-Vis titrations:

Procedure

The experimental procedure consisted of placing the solution of **1** in the cuvette, adding with a microsyringe a certain number of equivalents of pyrrolidine, shaking the cuvette and recording the absorption spectrum obtained in each case for every **1**:pyrrolidine ratio. As a result of the addition of pyrrolidine, the colour paled from orange to yellow.

Titration with pyrrolidine in 3:1:1 (v/v) DMSO/water/acetone

The absorption band centred at 390 nm decreases meanwhile another absorption band centred at 317 nm increases. There is an isosbestic point at 340 nm that disappears for excesses larger than 1 equiv of pyrrolidine.

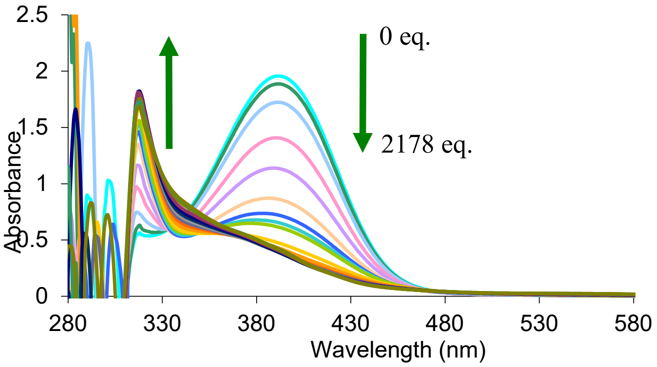

S36a

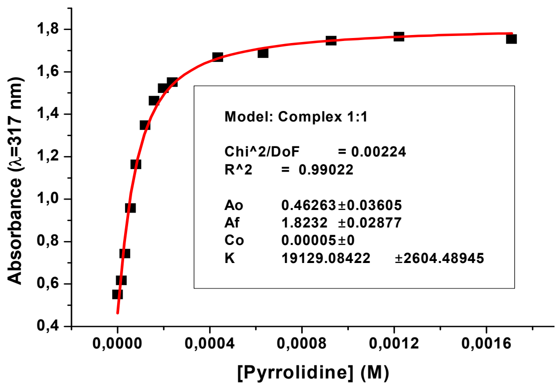

S36b

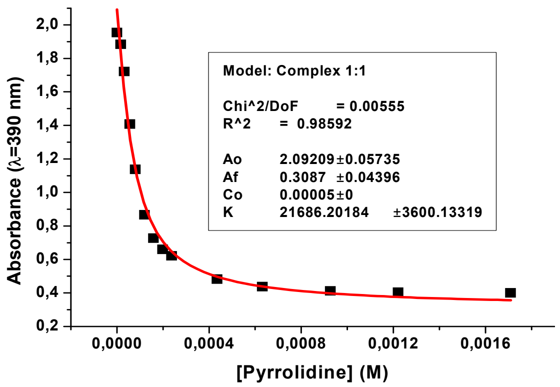

S36c

Figures S36a-c. (a) Absorption spectra and absorbance titration profiles fitted at (b) λ_{abs}= 317 nm or (c) λ_{abs}= 390 nm of 8a in 3:1:1 (v/v) DMSO/water/acetone with several pyrrolidine additions. (C_{8a}= 5.0 × 10^{-5} M).

Titration with pyrrolidine in DMSO

The absorption band centred at 405 nm decreases meanwhile another absorption band centred at 305 nm increases. There is an isosbestic point at 336 nm that disappears for excesses larger than 1 equiv of pyrrolidine.

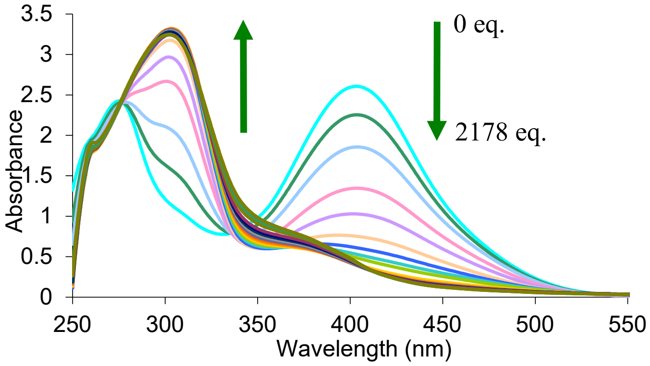

S37a

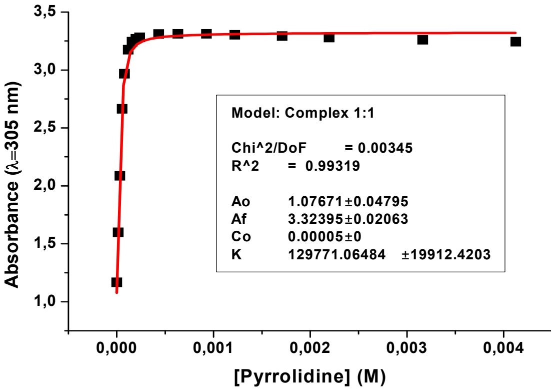

S37b

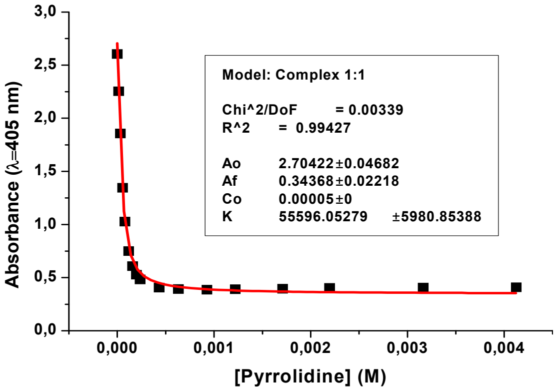

S37c

Figures S37a-c. (a) Absorption spectra and absorbance titration profiles fitted at (b) λ_{abs}= 305 nm or (c) λ_{abs}= 405 nm of 8a in DMSO with several pyrrolidine additions. (C_{8a}= 5.0 × 10^{-5} M).

Fluorescence measurements:

The detection of amines by the probe **1** can be followed by fluorescence measurements considering a two steps process, because the kinetic effect observed is very sharp. It consists of an initial extremely fast increase in the fluorescence followed by a very slow process of fluorescence intensity decay. The first step is related with the formation of a complex between the probe and the analyte and can be studied kinetically by using ultrafast techniques such as stopped flow or by fluorescent titrations measuring different ratios **1**:pyrrolidine immediately after the addition of the amine (t= 0). On the other hand, the second step corresponds to the decay of the complex, which can be studied by recording the fluorescence decay over time under pseudo-first order conditions (big excess of pyrrolidine): **1** + Pyrrolidine ↔ Complex → (Decomp.)

Pyrrolidine was selected as an example of secondary amine. The mechanisms was extrapolated to many other amines, such as the biogenic amines. Therefore, the results were compared between pyrrolidine, histamine, putrescine and cadaverine. Then, real samples of fish were studied upon decomposition and the possibility of using as an intelligent label was studied.

Fluorescent titration with pyrrolidine: The experimental procedure consisted of placing the solution of **1** in the cuvette, adding with a microsyringe a certain number of equivalents of pyrrolidine, shaking the cuvette and, immediately after de addition, recording the emission spectrum obtained in each case for every **1**:pyrrolidine ratio. The same procedure is repeated for each different **1**:pyrrolidine ratio. In order to compare with other amines, the spectra were also measured in DMSO solution.

a) Titration with pyrrolidine in 3:1:1 (v/v) DMSO/water/acetone

The emission band centred at 507 nm increases with the concentration of pyrrolidine. λ_{exc}= 340 nm.

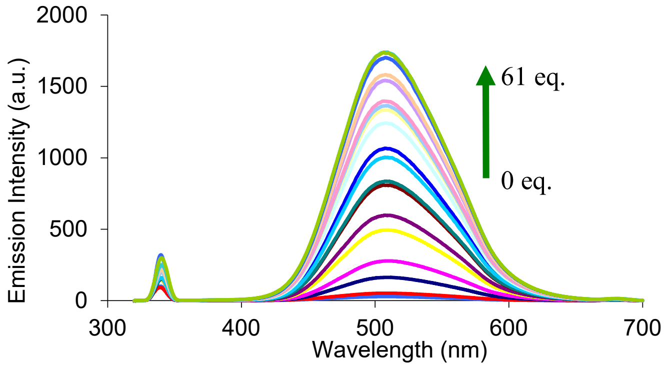

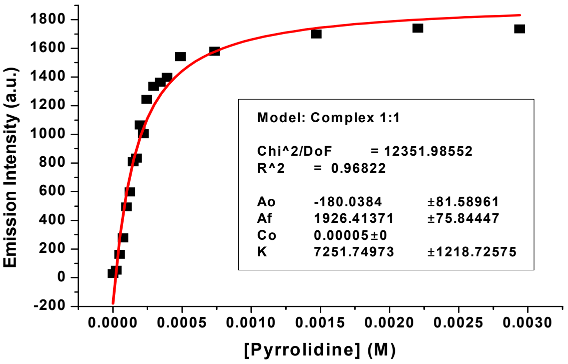

S38a **S38b**

Figures S38a-b. (a) Emission spectra and (b) emission intensity titration profile fitted at λ_{em}= 507 nm of 8a in 3:1:1 (v/v) DMSO/water/acetone with several pyrrolidine additions. (C_{8a}= 5.0 × 10^{-5} M).

b) Titration with pyrrolidine in DMSO:

The emission band centred at 507 nm increases with the concentration of pyrrolidine. λ_{exc}= 336 nm.

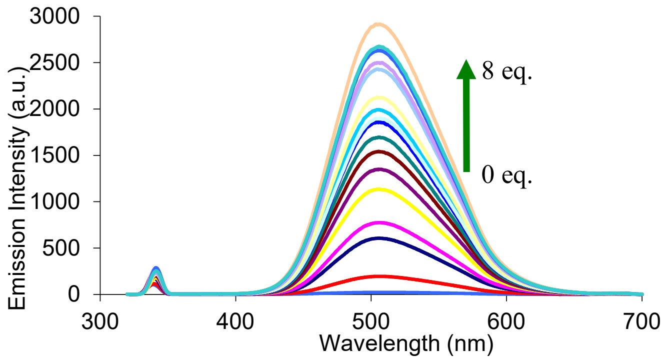

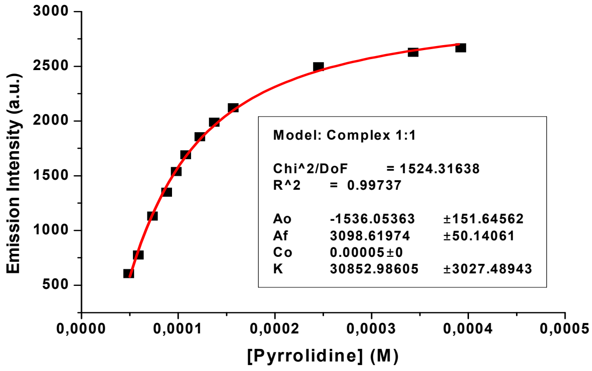

S39a **S39b**

Figures S39a-c. (a) Emission spectra and (b) emission intensity titration profile fitted at λ_{em}= 507 nm of 8a in DMSO with several pyrrolidine additions. (C_{8a}= 5.0 × 10^{-5} M).

Kinetic measurements with pyrrolidine

In this section, the evolution of the fluorescence over time of **1** in presence of high excesses of pyrrolidine is presented. The experimental procedure consisted of placing the solution of **1** in a fluorescence cuvette, adding with a microsyringe a certain number of equivalents of pyrrolidine, shaking the cuvette and recording the emission intensity emission at the maximum emission wavelength (λ_{exc}= 340 nm; λ_{em}= 506 nm; T= 25 ºC) for every **1**:pyrrolidine ratio. The curves obtained were fitted to first order rate law equation to obtain the rate constant (k).

$$Y_t = Y_\infty - (Y_\infty - Y_0)e^{kt}$$

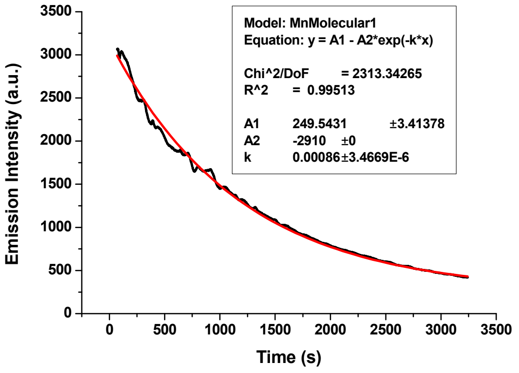

S40a

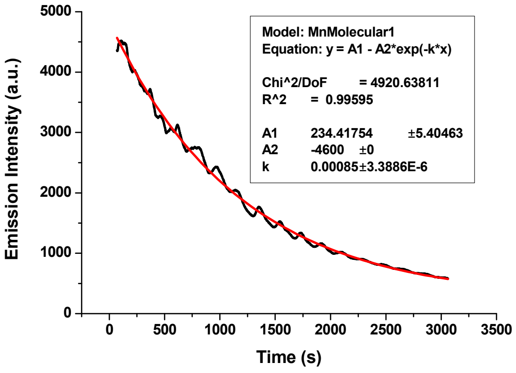

S40b

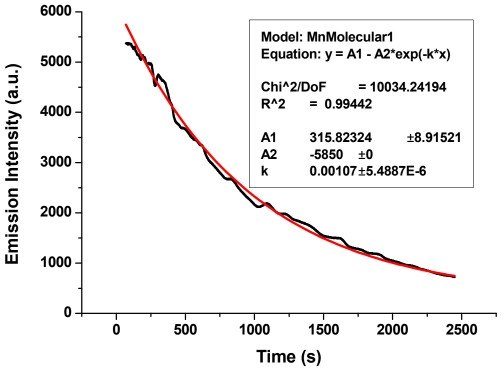

S40c

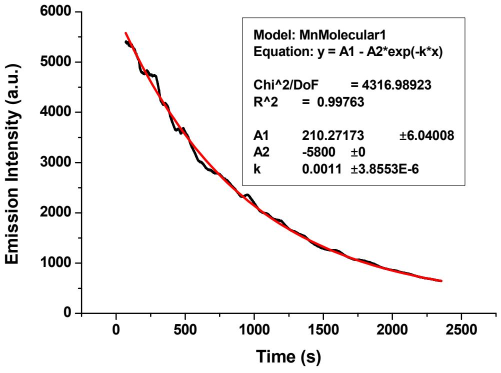

S40d

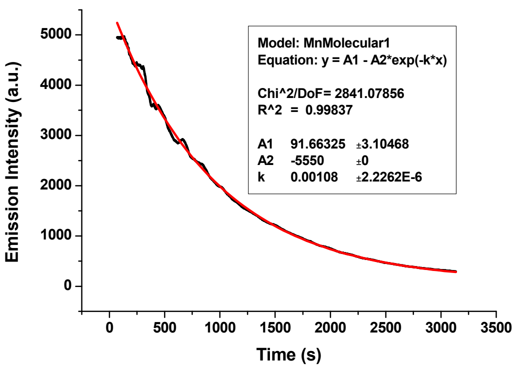

S40e

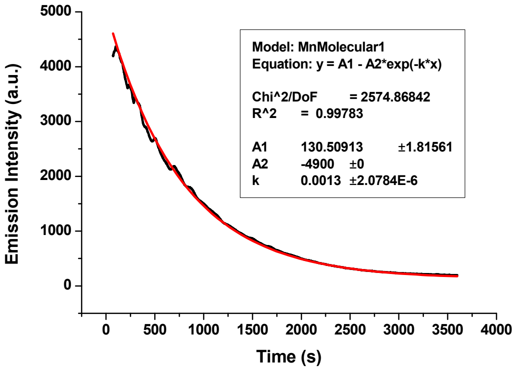

S40f

Figures S40a-h. Fluorescence measured (black line) and fitting curve (red line) for the fluorescence intensity decay process of a (a) 10:1, (b) 20:1, (c) 25:1, (d) 30:1, (e) 40:1 or (f) 50:1 (Pyrrolidine:1) concentration ratios in 3:1:1 (v/v) DMSO/water/acetone. (C_{8a}= 5.0 × 10^{-5} M).

$C_{Pyrrolidine}$:C_1	A1	A2	k	R^2
10:1	249.54	-2910	0.00086	0.995
20:1	234.42	-4600	0.00085	0.996
25:1	315.82	-5890	0.00107	0.994
30:1	210.27	-5800	0.00110	0.998
40:1	91.66	-5550	0.00108	0.998
50:1	130.51	-4900	0.00130	0.998

Table 1. Parameters obtained by the fitting of each Pyrrolidine:1 concentration ratios.

Plotting the rate constants calculated versus pyrrolidine concentration allowed us to obtain the equilibrium constant:

$$k_{v,obs} = k_1([Pyr.]_0) + k_2$$

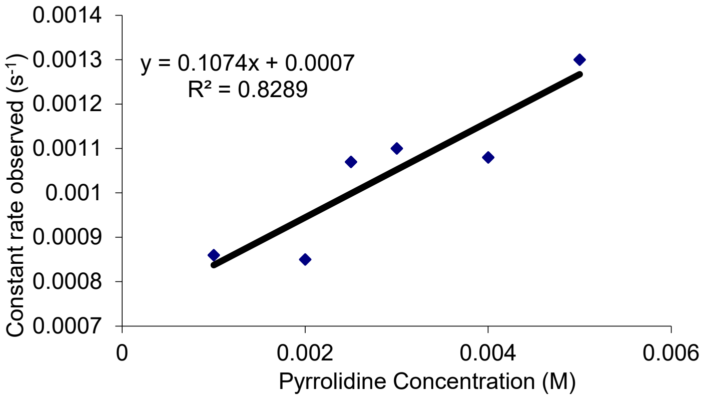

Figure S41. Representation of the rate constant observed versus pyrrolidine concentration.

According to these results and after validating the regression by using least median of squares, a mathematical tool to eliminate outliers, an equilibrium constant of 147.16 ± 49.14 M^{-1} was achieved for the decomposition process taking place after the **1**:Pyrrolidine complex formation.

Detection of biogenic amines:

Quantum Yield Measurements:

Quantum yields was measured for **1** in the presence of pyrrrolidine, used as reference, and histamine, putrescine and cadaverine. **1** was dissolved in DMSO, the reference was quinine sulfate in H_2SO_4 0.05M.

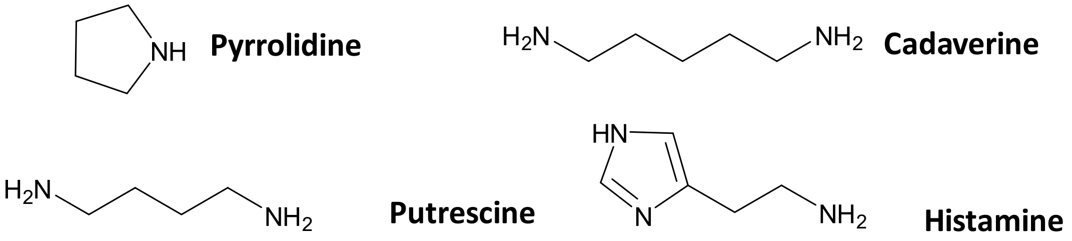

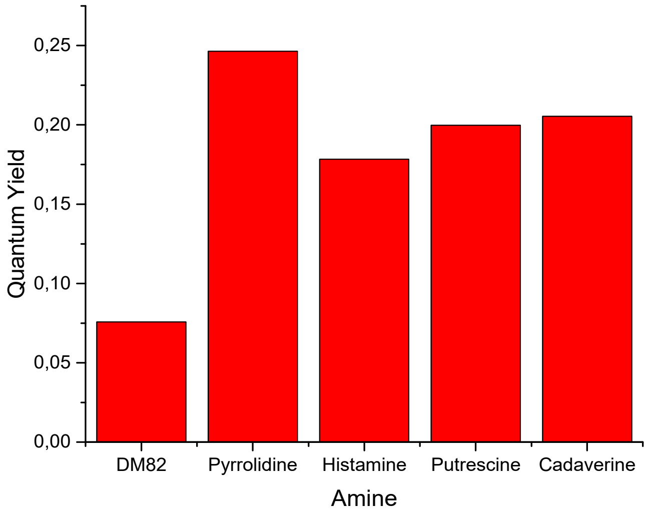

	Φ
**1**	0.08
**Pyrrolidine**	0.25
**Histamine**	0.18
**Putrescine**	0.20
**Cadaverine**	0.21

Figure S42. Quantum Yield of different combinations between 1 and amines in DMSO, comparation between: pyrrolidine, histamine, putrescine, cadaverine and without amine.

Measuring fish decay:

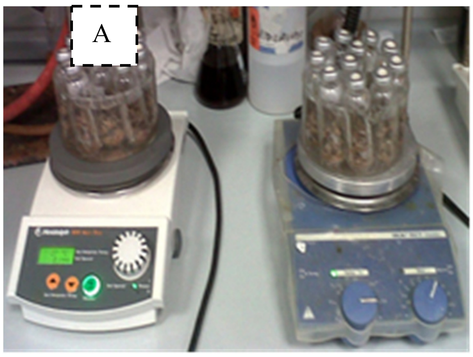

B

Figure S43. Experimental procedure for the incubation of samples at 25 ºC.

The process was repeated three times and the results combined (after normalizing to 25 days result). The combination gives an exponential increase in the amines produced by the fish at 25 ºC. This increase starts to be noticeable after 3-5 days, since that point the presence of biogenic amines is clear.

Measuring in supported probe:

Apart from the solution it was tested by the same way in gel, by this process, rotten fish is heated and the gas extracted to a vial that contains the gel sample:

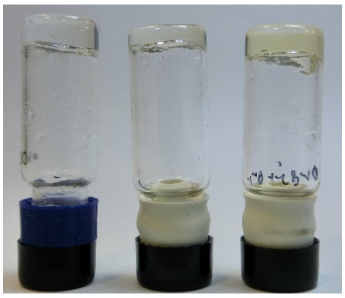

Figure S44. Samples of the gel 3.5 mg in 0.5 ml of DMSO (left), gel 4 mg in 0.5 ml of DMSO 10^{-5} M 1 (middle) and gel 4 mg in 0.5 ml of DMSO $5 \cdot 10^{-5}$ M 1.

With the gel, the presence of amines in fish samples was tested by the way showed in the next picture:

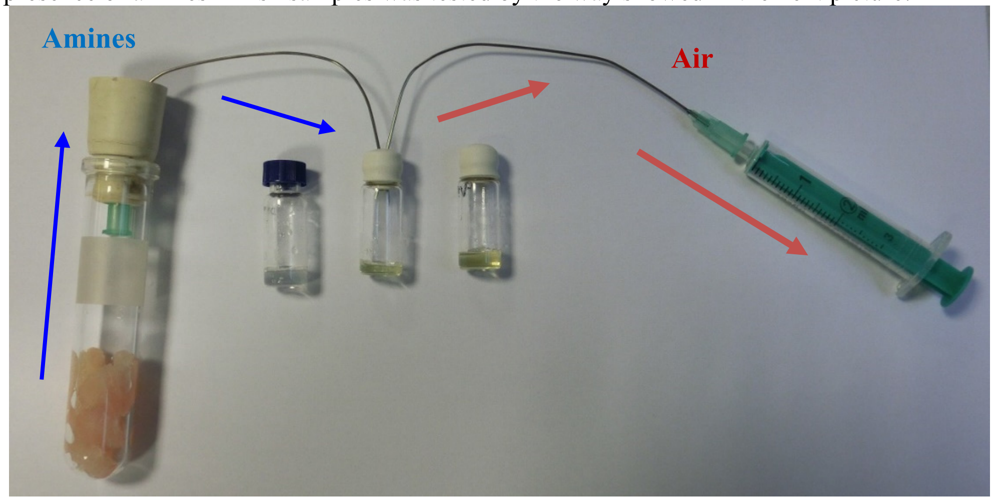

Figure S45. Mechanism of amines injection for the testing of the gel.

Quantum Chemical Calculations:

Geometric structure of compound **1**·was optimized at the B3LYP/6-31G* level, in which the Becke three parameter functional[1] and the Lee-Yang-Parr correlation functional were used,[2] implemented in the Gaussian 03 (Revision C.02) program suite.[3]

Coordinates of the stationary point

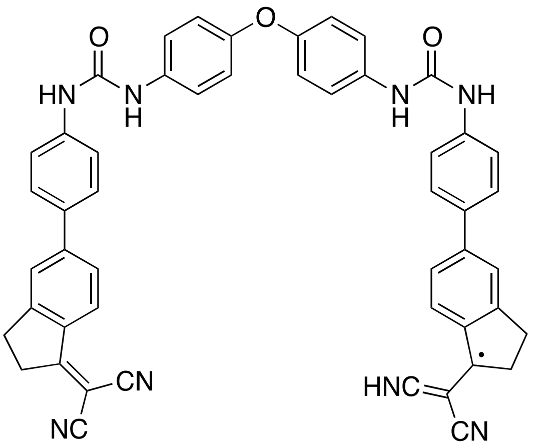

Figure S45

Standard orientation

Center Number	Atomic Number	Atomic Type	Coordinates (Angstroms) X	Y	Z
1	8	0	-2,778461	-3,165046	2,292866
2	6	0	-3,914468	-3,494624	1,585815
3	6	0	-5,061995	-3,754133	2,339671
4	6	0	-3,98107	-3,511138	0,189978
5	6	0	-6,269366	-4,011715	1,699281
6	1	0	-4,995048	-3,736841	3,422708
7	6	0	-5,186478	-3,80215	-0,446525
8	1	0	-3,092538	-3,312159	-0,399175
9	6	0	-6,345391	-4,043904	0,300269
10	1	0	-7,164559	-4,19591	2,287841
11	1	0	-5,223791	-3,833723	-1,529584
12	6	0	-1,521266	-3,439017	1,776073
13	6	0	-0,559113	-2,434343	1,865061
14	6	0	-1,173358	-4,693261	1,271691
15	6	0	0,743997	-2,688238	1,449429
16	1	0	-0,837665	-1,465683	2,267346
17	6	0	0,128345	-4,948506	0,844221
18	1	0	-1,91994	-5,4793	1,216651
19	6	0	1,103903	-3,942406	0,932059
20	1	0	1,490499	-1,900276	1,525979
21	1	0	0,401383	-5,921114	0,460115
22	7	0	2,442347	-4,108409	0,51984
23	1	0	3,044594	-3,311941	0,680215
24	7	0	-7,598755	-4,370085	-0,291839
25	1	0	-8,063833	-5,180482	0,100895

26	6	0	3,047809	-5,256186	0,054517
27	6	0	-8,048404	-4,163619	-1,587
28	8	0	2,499018	-6,344217	-0,060682
29	8	0	-8,7968	-4,961247	-2,135032
30	7	0	4,396548	-5,119224	-0,282577
31	1	0	4,829104	-6,026015	-0,401416
32	7	0	-7,621203	-3,018606	-2,261869
33	1	0	-7,890518	-3,082334	-3,236442
34	6	0	-7,363032	-1,712212	-1,798095
35	6	0	-6,778983	-0,805171	-2,696731
36	6	0	-7,693433	-1,265051	-0,51045
37	6	0	-6,528234	0,507117	-2,318744
38	1	0	-6,506869	-1,142399	-3,693912
39	6	0	-7,419929	0,04589	-0,137131
40	1	0	-8,177179	-1,935562	0,188995
41	6	0	-6,832882	0,964454	-1,024244
42	1	0	-6,050855	1,175322	-3,029341
43	1	0	-7,709201	0,372595	0,857455
44	6	0	5,243234	-3,99108	-0,274329
45	6	0	6,539469	-4,10671	0,251566
46	6	0	4,85179	-2,759626	-0,825293
47	6	0	7,409196	-3,023306	0,237938
48	1	0	6,853899	-5,049896	0,691092
49	6	0	5,722326	-1,673217	-0,810347
50	1	0	3,883295	-2,667516	-1,307534
51	6	0	7,021089	-1,773409	-0,281127
52	1	0	8,395268	-3,137123	0,67851
53	1	0	5,402449	-0,741828	-1,268355
54	6	0	-6,544171	2,355332	-0,612299
55	6	0	-6,167106	2,652554	0,710362
56	6	0	-6,638225	3,418684	-1,539736
57	6	0	-5,897015	3,962228	1,08237
58	1	0	-6,05821	1,848737	1,433136
59	6	0	-6,365471	4,729932	-1,181838
60	1	0	-6,960426	3,207837	-2,554579
61	6	0	-5,988827	5,013905	0,144932
62	1	0	-6,456292	5,518319	-1,919446
63	6	0	7,941025	-0,615889	-0,276572
64	6	0	7,452218	0,698945	-0,103582
65	6	0	9,331921	-0,792675	-0,442603
66	6	0	8,319193	1,774925	-0,100562
67	1	0	6,389532	0,858982	0,060682
68	6	0	10,215408	0,277425	-0,442821
69	1	0	9,721727	-1,794002	-0,60144
70	6	0	9,718768	1,589866	-0,273489
71	1	0	11,276094	0,090792	-0,570414
72	6	0	-5,468101	4,467832	2,438645

73	1	0	-6,200773	4,212415	3,213118
74	1	0	-4,518134	4,015803	2,748324
75	6	0	-5,337506	5,99961	2,251641
76	1	0	-6,034188	6,553765	2,891708
77	1	0	-4,338132	6,369821	2,506425
78	6	0	-5,648038	6,27023	0,789521
79	6	0	-5,586994	7,534489	0,256621
80	6	0	-5,221049	8,639774	1,088473
81	7	0	-4,919574	9,520377	1,788384
82	6	0	-5,868452	7,835914	-1,111404
83	7	0	-6,095977	8,083888	-2,22634
84	6	0	7,995792	3,240529	0,088698
85	1	0	7,317345	3,60372	-0,692932
86	1	0	7,490004	3,418734	1,045164
87	6	0	9,372792	3,957531	0,024847
88	1	0	9,595817	4,49588	0,955858
89	1	0	9,404587	4,714453	-0,769825
90	6	0	10,379999	2,852934	-0,224639
91	6	0	11,779366	3,137245	-0,386579
92	6	0	12,241245	4,488673	-0,231906
93	7	0	12,595552	5,590076	-0,105183
94	6	0	12,743137	2,253263	-0,737074
95	7	0	13,653265	1,449887	-0,858178
96	1	0	13,985089	1,219513	-1,802056

1 A. D. Becke, *J. Chem. Phys.* **1993**, *98*, 5648-5652

2 C. T. Lee, W. T. Yang, R. G. Parr, *Phys. Rev. B* **1988**, *37*, 785-789.

3 M. J. Frisch, G. W. Trucks, H. B. Schlegel, G. E. Scuseria, M. A. Robb, J. R. Cheeseman, J. A. Montgomery, Jr., T. Vreven, K. N. Kudin, J. C. Burant, J. M. Millam, S. S. Iyengar, J. Tomasi, V. Barone, B. Mennucci, M. Cossi, G. Scalmani, N. Rega, G. A. Petersson, H. Nakatsuji, M. Hada, M. Ehara, K. Toyota, R. Fukuda, J. Hasegawa, M. Ishida, T. Nakajima, Y. Honda, O. Kitao, H. Nakai, M. Klene, X. Li, J. E. Knox, H. P. Hratchian, J. B. Cross, C. Adamo, J. Jaramillo, R. Gomperts, R. E. Stratmann, O. Yazyev, A. J. Austin, R. Cammi, C. Pomelli, J. W. Ochterski, P. Y. Ayala, K. Morokuma, G. A. Voth, P. Salvador, J. J. Dannenberg, V. G. Zakrzewski, S. Dapprich, A. D. Daniels, M. C. Strain, O. Farkas, D. K. Malick, A. D. Rabuck, K. Raghavachari, J. B. Foresman, J. V. Ortiz, Q. Cui, A. G. Baboul, S. Clifford, J. Cioslowski, B. B. Stefanov, G. Liu, A. Liashenko, P. Piskorz, I. Komaromi, R. L. Martin, D. J. Fox, T. Keith, M. A. Al-Laham, C. Y. Peng, A. Nanayakkara, M. Challacombe, P. M. W. Gill, B. Johnson, W. Chen, M. W. Wong, C. Gonzalez, J. A. Pople, Gaussian 03, Revision C.02, Gaussian, Inc., Wallingford CT, **2004**.

Description of the molecular orbitals

SOMO:

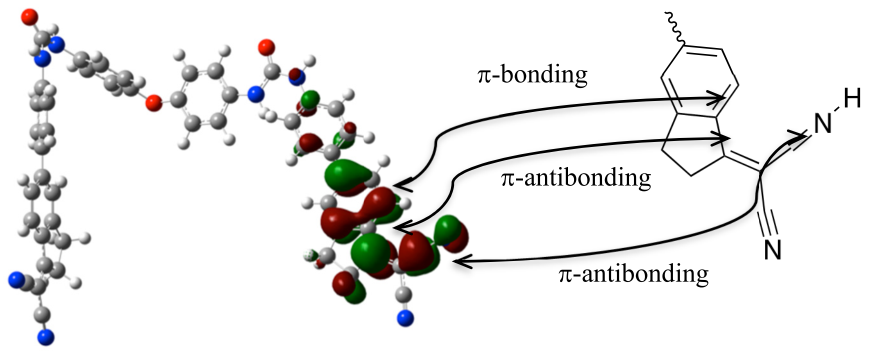

Figure S46: Single Occupied Molecular Orbital (SOMO) has mainly π-antibonding character located over the C=N bond of the protonated nitrogen and over the 1-indene carbon atom and the aryl group, as well as π-bonding over the aryl group bonded to the five members ring. The molecular orbital has slight contributions of σ-bonding interactions between hydrogen atoms and the former nitrile nitrogen atom and on carbon atom of the five membered ring.

SOMO+1:

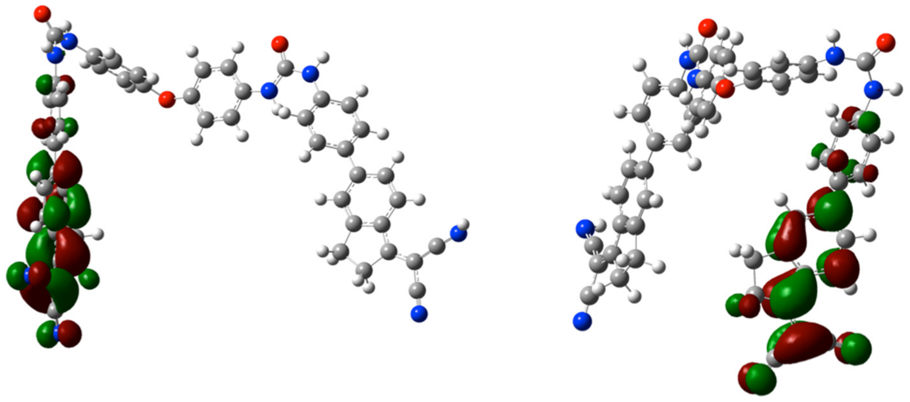

Figure S47: SOMO+1 is located on the arm of the molecule not affected by the formation of the radical. It has mainly π-antibonding character located over the aryl fragment near to the nitrile groups, and π-antibonding character in the nitrile groups. Nevertheless, this orbital has slight π-bonding character in the C-C interactions of the nitrile groups and the 1-indene carbon atom with the aryl fragment.

SOMO-1:

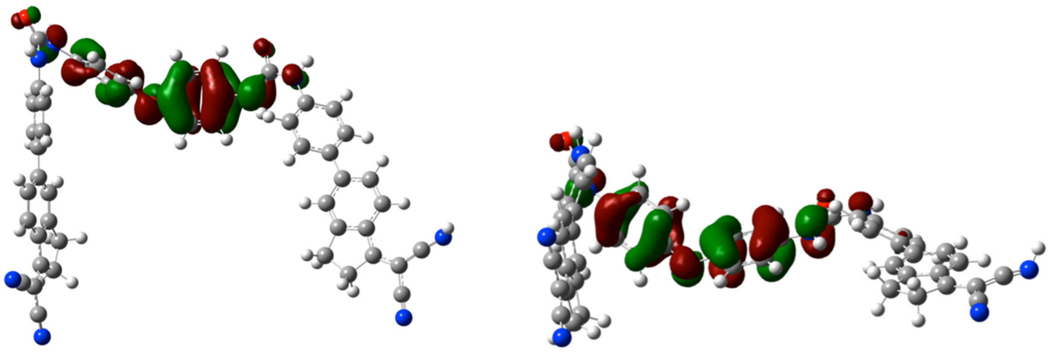

Figure S48: SOMO-1 is a π-bonding molecular orbital delocalized over the phenyl rings directly bonded to the ether oxygen atom and with slight π-antibonding interactions with a p orbital of this oxygen atom and with p orbitals of the urea nitrogen atoms.

SOMO-2:

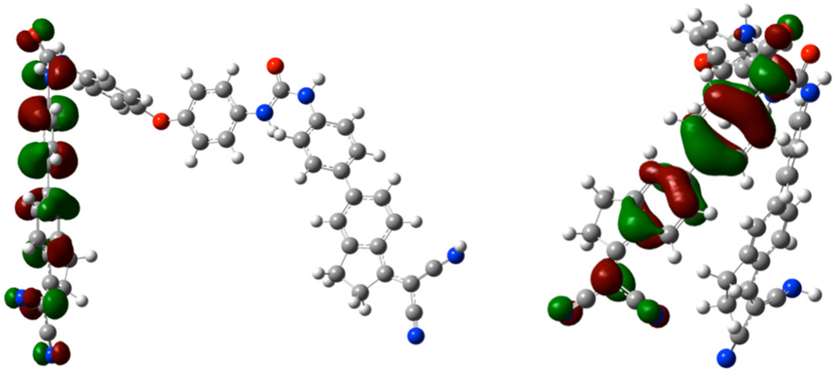

Figure S49: SOMO-2 is a π-bonding molecular orbital delocalized over the biphenyl rings of the arm not affected by the radical. There are also small contributions of p orbitals of the nitrile nitrogen atoms, the 1-indene exocyclic carbon atom, the oxygen atom and the nitrogen atom of the urea directly bonded to the biphenyl fragment.

SOMO-3:

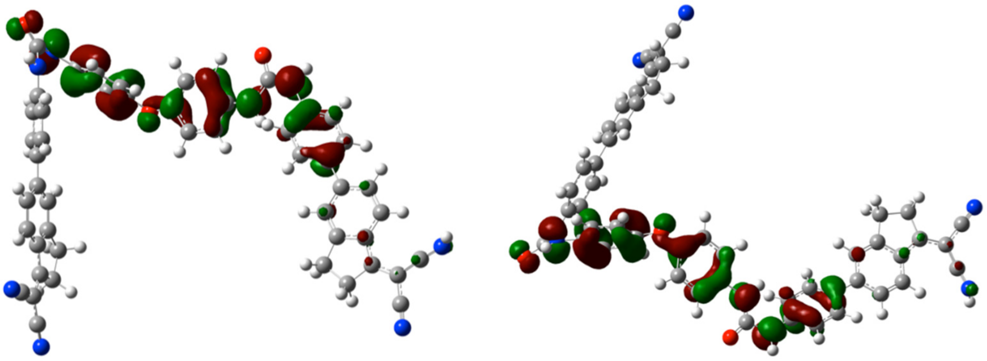

Figure S50: SOMO-3 is a π-bonding molecular orbital extended over the phenyl rings bonded to the ether oxygen. There is also participation of a p orbital of the oxygen ether. The molecular orbital has different participation of orbitals of atoms of each arm of the ether. In the arm not affected by the radical there is participation of the nitrogen atom of the urea fragment directly bonded to the phenyl ring of the ether and a p orbital of the oxygen atom of this fragment urea. In the arm affected by the radical, there is participation of p orbitals of both nitrogen atoms of the fragment urea and a π-bonding participation of the phenyl ring directly bonded to the urea fragment.
